# Regional thermal variation in a coral reef fish

**DOI:** 10.1093/conphys/coae058

**Published:** 2024-08-13

**Authors:** Elliott Schmidt, Jennifer M Donelson

**Affiliations:** College of Science and Engineering and ARC Centre of Excellence for Coral Reef Studies, James Cook University, Townsville, 4811, Australia; ARC Centre of Excellence for Coral Reef Studies, James Cook University, Townsville, Australia; College of Science and Engineering and ARC Centre of Excellence for Coral Reef Studies, James Cook University, Townsville, 4811, Australia; ARC Centre of Excellence for Coral Reef Studies, James Cook University, Townsville, Australia

**Keywords:** Damselfish, intraspecific variation, latitudinal gradient, temperature, physiology, adaptation

## Abstract

How species respond to climate change will depend on the collective response of populations. Intraspecific variation in traits, evolved through genetic adaptation and phenotypic plasticity, can cause thermal performance curves to vary over species’ distributions. Intraspecific variation within marine species has received relatively little attention due to the belief that marine systems lack dispersal barriers strong enough to promote locally adapted traits. Here we show that intraspecific variation is present between low- and high-latitude populations of a coral reef damselfish (*Acanthochromis polyacanthus*). Co-gradient variation was observed when examining aerobic physiology across a thermal gradient that reflected mean summer temperatures of high- and low-latitude regions, as well as projected future ocean temperatures (i.e. 27, 28.5, 30, 31.5°C). Whilst thermally sensitive, no significant differences were observed between high- and low-latitude regions when measuring immunocompetence, haematocrit and anaerobic enzyme activity. The presence of co-gradient variation suggests that dispersal limitations in marine systems can promote local adaptive responses; however, intraspecific variation may not be ubiquitous amongst traits. Identifying locally adapted traits amongst populations remains necessary to accurately project species responses to climate change and identify differences in adaptive potential.

## Teaser

This study investigated the thermal performance of several traits from damselfish populations in two different regions on the Great Barrier Reef (low- and high-latitude). Oxygen consumption rates and aerobic enzyme activity at elevated temperatures were found to differ between the regions, while immunocompetence, haematocrit, and anaerobic enzyme activity were similar.

## Abbreviations

(MO_2rest_): —Resting oxygen consumption
(MO_2max_): —Maximum oxygen consumption
AAS: —Absolute aerobic scope
(LDH): Lactate dehydrogenase
(CS): Citrate synthase

## Introduction

The response of species to climate change is determined by the collective response of populations (Bennett *et al.*, 2019; McKenzie *et al.*, 2021). How populations respond to environmental change can vary over geographic and environmental gradients, due to variation in traits that have evolved via genetic adaptation and phenotypic plasticity (Sorte *et al.*, 2011; Des Roches *et al.*, 2018; Bennett *et al.*, 2019; Plumb *et al.*, 2020). Temperature conditions, particularly amongst ectotherms, are hypothesized to produce macro-biogeographical patterns that reflect thermal constraints on organisms’ biochemistry and physiology (Somero, 2010; Pereira *et al.*, 2017). Co-gradient variation across thermal clines, whereby genetic and environmental influences on phenotype are aligned (e.g. populations exposed to higher temperatures have higher optimal performance temperatures), has been demonstrated in a variety of taxa (plants: Aitken and Bemmels, 2016; Mahony *et al.*, 2020, insects: Hoffmann *et al.*, 2003; Barton *et al.*, 2014, crustaceans: Kuo and Sanford, 2009; Sorte *et al.*, 2011; Yampolsky *et al.*, 2014 and fish: see review by Conover *et al.*, 2009). However, optimal performance temperatures often do not follow the trajectory of environmental gradients (Conover *et al.*, 2009). Countergradient variation occurs when phenotypes possess greater plastic responses than phenotypic divergence between populations, or phenotypes diverge in the opposite direction of environmental gradients (Conover and Schultz, 1995; Schmid and Guillaume, 2017; Stamp and Hadfield, 2020). Countergradient variation has been recorded in several taxa (lizards: Angilletta *et al.*, 2004; Hodgson and Schwanz, 2019, turtles: Snover *et al.*, 2015 and fish: Gardiner *et al.*, 2010); however, the extent to which phenotypic plasticity and genetic differentiation contribute to countergradient variation differs (Stamp and Hadfield, 2020).

Population responses to warming temperatures will depend on their occupied thermal niche. Low-latitude environments characterized by stable temperatures near physiological maximums favour specialized (narrow) thermal niche breadths that primarily evolved through genetic adaptation (i.e. selection for particular phenotypes) rather than plasticity—Climate Variability Hypothesis (Janzen, 1967; Stevens, 1989; *but see*Overgaard *et al.*, 2011; Chiono and Paul, 2023). Narrow thermal niche breadths, limited plasticity, and evidence of hard ceilings for upper thermal tolerance (Gunderson and Stillman, 2015; Sandblom *et al.*, 2016; Morgan *et al.*, 2020) suggest that low-latitude populations are more vulnerable to shifting temperatures than high-latitude populations (Stillman, 2003; Deutsch *et al.*, 2008; Tewksbury *et al.*, 2008; Somero, 2010; Sunday *et al.*, 2011). High-latitude populations that experience variable environmental conditions are predicted to retain greater benefits from phenotypic plasticity than populations living in thermally stable environments (Janzen, 1967; Stevens, 1989); nonetheless, empirical evidence remains scarce (but see, Molina-Montenegro and Naya, 2012; Naya *et al.*, 2012; Donelson *et al.*, 2019). Wider thermal niche breadths have been reported in high-latitude populations (Sunday *et al.*, 2011; Shah *et al.*, 2017; Stuart-Smith *et al.*, 2017; McKenzie *et al.*, 2021), however, heat-tolerant phenotypes present in low-latitude populations may be unattainable within high-latitude populations (Kelly *et al.*, 2012). Individual populations may therefore possess thermal niches that are narrower than the species as a whole (Kelly and Griffiths, 2021).

Intraspecific variation in thermal performance within marine systems has not received the same attention as terrestrial systems, despite marine organisms having greater confinement to thermal tolerance limits (Sanford and Kelly, 2011; Sunday *et al.*, 2011; Pinsky *et al.*, 2019; Lenoir *et al.*, 2020). Within terrestrial systems local adaptation is already being incorporated into conservation considerations to prepare organisms for projected climate change scenarios (Aitken and Whitlock, 2013; Aitken and Bemmels, 2016; Liepe *et al.*, 2016; Bazzicalupo *et al.*, 2023). Marine systems hereinto have been demographically viewed as well-connected networks where locally adapted traits are expected to be overwhelmed by gene flow. However, a growing body of evidence suggests that oceanographic features, life history traits and larval dispersal ability can act as challenges to gene flow and promote local adaptation (Jones *et al.*, 1999; Swearer *et al.*, 2002; Sanford and Kelly, 2011). Evidence of local adaptation and how it impacts the ability to predict species responses to climate change has been demonstrated amongst marine crustaceans (Stillman, 2002; Kuo and Sanford, 2009; Sorte *et al.*, 2011; Kelly *et al.*, 2012; Pereira *et al.*, 2017; Sasaki and Dam, 2019) and corals (van Oppen *et al.*, 2014); yet few studies broach the topic amongst marine fish.

Thermal intraspecific variation in marine fishes may differ depending on larval dispersal ability and local environmental conditions; therefore, broadscale geographical patterns such as the climate variability hypothesis and co-/countergradient variation are unlikely to be universally applicable (Calosi *et al.*, 2008; Pereira *et al.*, 2017; Sasaki and Dam, 2019). A case study comparing low- and high-latitude populations of coral trout (*Plectropomus leopardus*), a species with a pelagic larval stage and high level of population connectivity (via spatial and temporal variation in larval recruitment; Van Herwerden *et al.*, 2009; Taboun *et al.*, 2021), found no significant differences in physiological metrics between populations (Pratchett *et al.*, 2013). However, patterns of countergradient variation and genetic distinctness have been identified amongst marine fish species with high- (*Gadus morhua*; Marcil *et al.*, 2006) and low-dispersal (*Acanthochromis polyacanthus*; Gardiner *et al.*, 2010; Donelson and Munday, 2012) ability between populations. The lack of uniformity in broadscale geographic patterns amongst marine fish necessitates the examination of population-based responses.

Genetic differentiation and intraspecific variation in thermal physiology of the coral reef damselfish *A. polyacanthus* is evident; however, existing physiological studies provide a coarse understanding of differences between populations. The inability for *A. polyacanthus* to disperse through water channels greater than ~10 m has created a genetically isolated and heterogenous mixture of populations (reefs) that are strongly influenced by founder effects (Doherty *et al.*, 1994; Planes *et al.*, 2001; Miller-Sims *et al.*, 2008). Between different regions, and even neighbouring reefs, populations display divergence at the nuclear level and lack the ability to exchange genes in the absence of connecting shallow channels (Doherty *et al.*, 1994; Planes *et al.*, 2001). Hereinto, physiological data from high-latitude populations emanates from a single lagoonal population that experiences high levels of diurnal and seasonal temperature fluctuations compared to most reef systems (i.e. Heron Island; Gardiner *et al.*, 2010; Donelson and Munday, 2012). When comparing wild-sourced *A. polyacanthus* from Heron Island and low-latitude populations (i.e. Lizard Island, Palm Island) oxygen consumption rates at elevated temperatures were found to be similar between regions (Donelson and Munday, 2012) or more efficient amongst fish from Heron Island (Gardiner *et al.*, 2010)—countergradient variation. Results on equatorial *A. polyacanthus* populations (Papua New Guinea), following comparative methods to Gardiner *et al.* (2010), found complementary evidence that optimal temperatures for oxygen consumption were similar between equatorial, low-latitude and high-latitude populations (Rummer *et al.*, 2014). Populations from equatorial or low-latitude regions may therefore be more susceptible to warming ocean temperatures than high-latitude populations as they are already living closer to their thermal limits (Rummer *et al.*, 2014). However, these results come from the sampling of just four populations across *A. polyacanthus*’s range, and it remains unclear how representative, and applicable, these results are to neighbouring populations or throughout the species range.

The objective of this study was to increase the resolution of *A. polyacanthus’s* thermal landscape and allude to a finer understanding of existing intraspecific variation within marine environments. Thermal performance curves of key physiological traits in *A. polyacanthus* were compared using three different reefs amongst two regions of the Great Barrier Reef (GBR), low latitude (~Cairns) and high latitude (~Mackay), that experience different thermal profiles. Based on previous studies, we hypothesized that high-latitude populations that experience greater thermal variation will possess greater physiological performance at elevated temperatures than low-latitude populations (i.e. countergradient variation). At elevated temperatures, fish from higher latitudes were expected to outperform fish from low latitudes when measuring physiologically informative metrics including aerobic physiology (i.e. oxygen consumption), immunocompetence, haematocrit, and enzyme activity. Tested physiological metrics are associated with energetic demanding processes that have previously been identified as being sensitive to environmental temperatures (Supplementary Table S1). Additionally, co-gradient variation remains a valid alternative hypothesis considering previously demonstrated genetic differentiation, lack of variability in studied populations and the unique nature of the previously tested Heron Island population.

## Materials and Methods

This research was completed under James Cook University ethics approval A2764.

### Study species

The tropical damselfish, *A. polyacanthus* (Bleeker, 1855), ranges from the Philippines to the southern end of the GBR (~15°N to ~23°S; Allen, 1991). *Acanthochromis polyacanthus* populations are hypothesized to have propagated throughout the Indo-Pacific proceeding the Pleistocene (2.6 Ma–11.7 ka) as rising sea levels reestablished dispersal corridors between reefs (Van Herwerden and Doherty, 2006; Ludt and Rocha, 2015). However, such dispersal corridors dissipated as sea levels reached present-day conditions (Miller-Sims *et al.*, 2008). *Acanthochromis polyacanthus* lacks a pelagic larval development period, instead performing parental care during embryonic and early life development, in socially monogamous pairs, until fry are large enough to disperse into the surrounding habitat (Robertson, 1973). Dispersal is limited to adjacent reefs separated by depths less than ~10 m, creating conditions where reefs that are not connected by shallow channels are genetically isolated and differentiated from one another (electrophoresis, Doherty *et al.*, 1994; microsatellite markers, Miller-Sims *et al.*, 2008). *Acanthochromis polyacanthus* are ideal for examining local adaptation in marine environments as they possess a broad geographic distribution, across thermally variable environments, where gene flow is limited.

### Sampling

**Figure 1:**
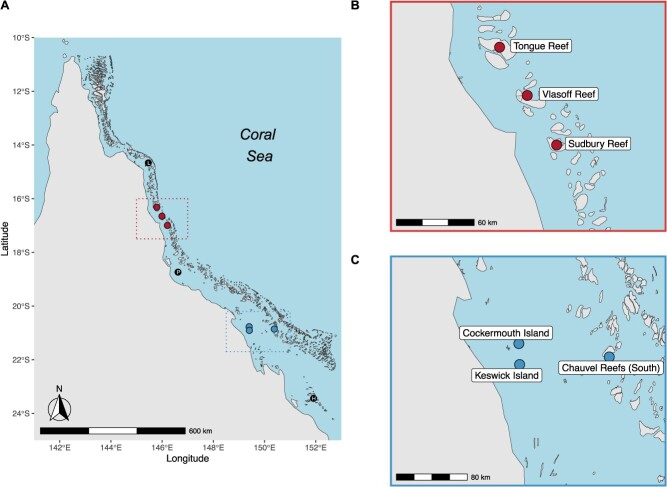
Maps outlining A) low- and high-latitudinal regions that fish were collected from across the Great Barrier Reef. Black points represent sites where fish were sampled in previous research, including Lizard Island (L), Palm Island (P) and Heron Island (H). Insert B) provides a zoomed-in perspective of the low-latitude region that was made up of fish from three different reefs including Sudbury Reef, Tongue Reef and Vlasoff Reef. Insert C) provides a zoomed-in perspective of the high-latitude region that is made up of two inshore islands, Cockermouth and Keswick Island, and one offshore reef, Chauvel Reef (southern).

Adult *A. polyacanthus* were collected via professional collectors from June to December 2021 from six different reefs and two different regions (low- and high-latitude) that were absent of shallow channels, which would have allowed dispersal between populations. Three reefs from low-latitude locations were sampled, including Tongue Reef (−16.341°, 145.773°; *n = 8*), Vlassof Cay (−16.657°, 145.990°; *n = 10*) and Sudbury Reef (−16.996°, 146.202°; *n = 11*). High-latitude sites included Cockermouth Island (−20.772°, 149.390°; *n = 10*), Keswick Island (−20.908°, 149.406°; *n = 6*) and Chauvel Reef (−20.863°, 150.363°; *n = 10;*Fig. 1). Low- and high-latitude collection sites are separated by ~400 km (spanning ~5° in latitude). In total, 55 fish were sampled over the duration of the experiment (Supplementary Table S2). Of the initial 55 fish, 38 completed all experimental assays including: resting oxygen consumption, maximum oxygen consumption, absolute aerobic scope, immunocompetence, haematocrit, and enzyme activity. Seventeen fish experienced mortality events at various stages of the experiment.

Adult fish were held in separate 52-l opaque aquariums (56 × 35 × 30 cm) inside an environmentally controlled aquarium room at the Marine and Aquaculture Research Facility at James Cook University (Townsville, Australia). Each aquarium contained a shelter (half a terra-cotta pot), constant aeration and water flow (2 l min^−1^) at set experimental conditions (see below). Fish were transferred to the experiment room that was used for trials on 25 May 2022. Respirometry and immunity trials occurred from 6 June to 17 August 2022. Tissue (enzymes) and blood (haematocrit) samples were collected on 1 September 2022, 2 weeks after respirometry and immunity trails concluded. A random number generator was used to determine the order and chamber fish were tested in.

### Thermal conditions

To understand local thermal conditions for reefs within low-latitude and high-latitude regions, temperature data was collected via the Australian Institute of Marine Science (AIMS) temperature logger data series. Temperature data was collected from loggers at depths of 7–15 m, for a subset of reefs (Supplementary Table S3) from each region (AIMS 2020; Supplementary Fig. S1). Experimental temperatures used for repeated oxygen consumption and immunocompetence thermal performance curves included the approximate daily mean summer temperature for both high-latitude (~27°C) and low-latitude (~28.5°C) regions, as well as 30 and 31.5°C. By mid-century, IPCC climate change scenarios project global surface temperature changes of +1.5°C by mid-century; two of these scenarios, SSP3–7.0 and SSP5–8.5, project > +3.0°C by 2100 (IPCC, 2021). For low-latitude populations 30 and 31.5°C represent projected mid- and end-of-century conditions; additionally, 31.5°C represents approximate present-day maximum summer temperatures. For high-latitude populations 28.5 and 30°C represent projected mid- and end-of-century conditions, with 30°C also representing approximate present-day maximum summer temperatures and 31.5°C representing a temperature that is presently not experienced. Testing began at the coolest temperature of 27°C. Fish were given at least 48 h to rest between aerobic physiology and immunocompetence trails. Once oxygen consumption and immunocompetence testing was completed for a given temperature, fish were warmed to the next temperature (+1.5°C), at a rate of +0.5°C day^−1^ for three consecutive days. Fish were then provided with an additional 5 days to adjust to the new temperature before the next sampling period began. Sampling periods took place over 4–5 days. This process was repeated for all testing temperatures.

### Aerobic physiology

Resting and maximum oxygen consumption were determined via intermittent flow respirometry. Chambers were 1.5 l in volume and custom built from PVC pipe and acrylic (Supplementary Fig. S2). The experimental setup consisted of two temperature-controlled aquaria (260 l), with continuous water exchange and aeration, each containing four submerged respirometry chambers placed in parallel. Chambers were opaque except for the lid, so that fish could not view each other, but light could still enter the chamber. Each respirometry chamber unit contained an independent brushless DC recirculation pump (flow rate 240 l h^−1^), vinyl tubing (composing ~1% of the total water volume) and an inline oxygen sensor probe (multichannel FireSting-O2, PyroScience GmbH, Aachen, Germany). Oxygen sensor probes were calibrated to 0% air, using sodium sulphite (Na_2_SO_3_)-saturated seawater, at the beginning of the experiment and when spot material was replaced. One hundred percent air calibrations were conducted at the beginning of each trial. During flush periods a pump (AQUAPRO, AP750LV; 750 l h^−1^) was used to flush each set of four chambers simultaneously. Heaters (2 kW) and temperature sensors (Semitec 103AT-11 IP67) were used to ensure that experimental temperatures remained within ±0.3°C of experimental temperature set points. Minimal background respiration was achieved through UV filtration, particle filtration (100-μm bag filters) and daily cleaning of equipment (bleach diluted to 200 ppm with fresh water). Fish were deprived of food for 18–24 h before respiration trials began (Chabot *et al.*, 2016). Trials were conducted in a fully lit room to eliminate metabolic costs associated with photoperiod (Chabot *et al.*, 2016).

Maximum oxygen consumption (MO_2max_) was used as a proxy for maximum metabolic rate (Norin and Clark, 2016). To achieve maximum oxygen consumption fish were placed in a swim tunnel for 10 min. During the initial 5-min interval, the speed of water flow through the swim tunnel was slowly increased until fish displayed a change in gait swimming behaviour, defined as a transitioning behaviour from predominately pectoral swimming to body/tail undulations (Supplementary Video 1). The speed of the swim tunnel that produced this intermediary transitional swimming behaviour was maintained for the second 5-min interval. Immediately after the 10-min swimming period, fish were collected by hand and transferred to a randomly selected respiration chamber. Pilot studies (unpublished data, Schmidt) determined that highest MO_2max_ levels were achieved with the immediate transfer of fish from the swim tunnel to respiration chambers, rather than including an intermediary air exposure period. Therefore, no extended air exposure time was included prior to fish being transferred into respiration chambers. The time between fish being placed in respiration chambers and data being recorded (i.e. start of the wait period) was <10 s. The MO_2max_ was measured over 30-s intervals via rolling regressions within the *‘*auto_rate’ function included in the R package ‘*respR*’ (v2.0.1; Harianto *et al.*, 2019). The steepest slope (highest oxygen consumption rate) with an *r^2^* threshold of 0.95 was used to determine MO_2max_. The MO_2max_ was measured prior to resting metabolic rate (MO_2rest_).

Fish were held in respirometry chambers for 3.5–6 h ($\overline{\mathrm{\mu}}$ = 4.67 h) to measure MO_2rest_. Measurement times for MO_2rest_ were based off previous experiments conducted on *A. polyacanthus* (Nilsson *et al.*, 2009; Donelson and Munday, 2012; Bernal *et al.*, 2018). Additionally, amongst small warm water fish, Rummer *et al.* (2014) found no deviation or continued decrease in oxygen consumption rates after 90 min of fish being placed in respiration chambers. Oxygen consumption was measured continuously over cycles consisting of a 15-s wait, 225-s measurement and 180-s flush period. Air percentage never dropped below <80% air saturation. Oxygen consumption rates were measured over a 220-s interval with an *r^2^* threshold of 0.95. The MO_2rest_ was measured by taking the mean of the lowest three oxygen consumption slopes. Background respiration was measured at the start of each trial by measuring oxygen consumption within empty chambers for at least three consecutive cycles. Background respiration levels typically accounted for <2% of measured oxygen usage rates and were therefore ignored. The mass of fish was measured at the end of all respiratory trials, after fish had been euthanized and patted dry with paper towels to avoid the inclusion of excess moisture. The mean fish-to-chamber volume ratio was 1:60 (Supplementary Fig. S3) but varied depending on the size of each fish. Oxygen consumption rates were converted from percent air saturation values to milligrammes per hour via the ‘*convert_rate*’ function within the R package *respR* (Harianto *et al.*, 2019). Absolute aerobic scope (AAS) was calculated by subtracting MO_2rest_ from MO_2max._

### Immune response

To test immunocompetence, subcutaneous phytohaemagglutinin injections were used to produce a (localized) cell-mediated response *in vivo* (e.g. inflammation, T-cell proliferation, infiltration of immune cells; Martin *et al.*, 2006; LaMonica *et al.*, 2021; pilot study on *A. polyacanthus* conducted by Donelson and Yasutake, 2024). Tissue swelling 24 h post-injection is mediated via complex immunological cascade. However, this tissue swelling is primarily driven via the congregation of leukocytes to the injection site (Martin *et al.*, 2006). Fish were injected in the caudal peduncle subcutaneously with 0.03 ml of phytohaemagglutinin (PHA; L8754 Sigma-Aldrich, 45 ug 10 ul^−1^) dissolved in phosphate buffer saline (PBS), made to a ratio of 1 mg PHA to 1 ml PBS. The immunocompetence of fish was determined by measuring the width of the injection area with pressure-sensitive callipers (Mitutoyo ABS Digimatic; accuracy 0.1 mm) pre-injection and ~18–24 h post-injection. The difference in localized swelling pre- and post-injection was used as a proxy for immunocompetence.

### Fish tissue sampling

Whole blood and white muscle tissue samples were collected 10 days after all oxygen consumption and immunocompetence trails were completed at the final testing temperature (31.5°C). Whole blood was collected from the caudal vein via heparin-coated 25-gauge surgical needles. Fish were then euthanized via cervical dislocation. White muscle tissue samples were dissected from tissue between the dorsal fin and lateral line. Once obtained, tissue samples were stored in liquid nitrogen and then transferred to a −80°C freezer.

### Haematocrit

Microcapillary tubes (75 mm Drummond Hemata-clad plain) were used to centrifuge blood samples at 10 000 rpm for 60 s to separate red cells from blood plasma. The proportion of blood volume occupied by red blood cells (haematocrit) was recorded by using a ruler to first measure the space of the microcapillary tube that was occupied by the total blood volume (packed red blood cells and blood plasma), followed by measuring the space occupied by packed red blood cells. Haematocrit scores were calculated using the following formula:


$$hematocrit=\frac{packed\ red\ blood\ cells}{total\ blood\ volume}$$

### Enzyme activity

White muscle tissue was used to examine the maximal enzyme activity of lactate dehydrogenase (LDH) and citrate synthase (CS). Testing temperatures of 20, 30, 40 and 50°C were used to measure enzyme activity and the associated thermal performance curve. White muscle tissue was used for measuring enzyme activity analysis because its anaerobic capacity has been shown to correlate to whole-organism oxygen consumption, it contributes to the largest fraction of body mass and plays an important role in high-speed burst swimming (Sullivan and Somero, 1980).

The protocol used to measure enzyme activity method used was adapted from previous studies (Thibault *et al.*, 1997; Seebacher *et al.*, 2003; Lang *et al.*, 2021). Samples were defrosted on ice. A sterile scalpel blade was used to extract a tissue sample (20–40 mg). Extracted tissue samples were homogenized via a microtube homogenizer (BeadBug 3, Benchmark Scientific, Model D1030-E) in a 1:10 dilution with a buffer consisting of 50 mmol l^−1^ 4-(2-hydroxyethyl)-1-piperazineethanesulfonic acid (HEPES), 1 mmol l^−1^ ethylenediaminetetraacetic acid (EDTA), 0.01% Triton X-100 and 99.99% Milli-Q water, and adjusted to pH 7.4 with sodium hydroxide (NaOH). A subset of homogenized tissue was extracted for LDH and CS. Homogenized tissue samples used for the LDH assay were centrifuged (Eppendorf Centrifuge 5424, Hamburg, Germany) at 150 rpm for <3 s. Homogenized tissue samples used for the CS assay were not centrifuged to allow mitochondria to be retained.

Absorbance readings were measured with a spectrophotometer every 2 s, with 20 readings over 13 min (UV5, Mettler-Toledo, Columbus, OH). Testing temperatures were maintained with a Loop L100 circulation thermostat (Lauda, Lauda-Königshofen, Germany). All samples were measured in triplicate and included a positive and negative control.

LDH was assayed at a final dilution of 1:200 in 0.5 mmol l^−1^ of β*-*nicotinamide adenine dinucleotide reduced disodium salt hydrate (NADH)-TRIS solution (pH 7.4) and 50 mmol l^−1^ of sodium-pyruvate-NADH-TRIS solution (pH 7.4). NADH absorbance was measured at a wavelength of 340 nm (Seebacher *et al.*, 2003). CS was assayed at a final dilution of 1:100 in 2 mmol l^−1^ 5,5′-dithobis-2-nitrobenzoic acid (DTNB)-ethanol solution, 12 mmol l^−1^ acetyl coenzyme A-lithium salt-Milli-Q solution and 50 mmol l^−1^ oxaloacetic acid-Tris solution (pH 8.0). DTNB absorbance was measured at a wavelength of 412 nm (Seebacher *et al.*, 2003; Blank *et al.*, 2004).

The mean slope was used to determine enzyme activity. Background activity was subtracted from sample absorbance slopes when background activity exceeded 5% of sample absorption levels. Absolute enzyme activity levels were calculated in units per milligramme tissue (U mg^−1^ tissue) using the following formula where: $\overline{A}$ represents the absolute mean absorption of tested sample in triplicate, $L$ represents the light path length (centimetre), $\varepsilon$ represents the molar absorptivity/extinction coefficient (M^−1^ cm^−1^), $c$ represents tissue sample concentration (mg/ml), and $V$ represents volume.


$$ \left| LDH/{CS}_{activity}\right|=\frac{\big(\overline{A}-{\overline{A}}_{background}\big)}{L\ast \varepsilon \ast c}\ast \frac{V_{assay}}{V_{sample}} $$

### Statistical analysis

Fish from different populations were grouped together based on whether they were collected from low- (Sudbury Reef, Tongue Reef and Vlassof Cay) or high-latitude (Cockermouth Island, Keswick Island and Chauvel Reef) reefs. From here onwards these groups will be referred to as low- or high-latitude regions. Generalized linear mixed effect models were used to test for differences in thermal performance curves associated with oxygen consumption, immunocompetence, and enzyme activity between low- and high-latitude populations (Supplementary Table S4). Model comparison and selection were performed using Akaike information criterion (AICc), Bayesian information criterion (BIC), and r-squared values (*see* software and packages section for more information). For thermal performance curves, model selection was used to determine if temperature should be modelled as a first, second or third order polynomial. Model selection was also used to compare alternative hypothesis-driven models (see ‘Software’ section in Methods for details). Statistical significance of independent variables and interactions was determined via likelihood-ratio chi-square values. Significant differences in *post hoc* analysis was determined by using estimated marginal means. Individual identification codes for each fish were used as a random factor due to repeated measures when modelling oxygen consumption, immunocompetence, and enzyme activity thermal performance curves.

All oxygen consumption models were run using a Gaussian distribution. To model oxygen consumption, including MO_2rest_, MO_2max_, and AAS, independent variables including latitude and temperature were modelled as fixed factors with an interaction term. Fish mass (centred) was positively related to oxygen consumption and therefore used as a covariate within oxygen consumption models. All oxygen consumption traits were modelled with temperature as a continuous second order polynomial. The model for MO_2rest_ included the additional covariate of testing runtime.

Immunocompetence was modelled as a function of latitude, temperature (third order polynomial) and their interaction term. Additionally, a gamma distribution (with a log-link function) was used instead of a Gaussian distribution. Fish mass was not included as a co-variate within the immunocompetence model.

Haematocrit was modelled via a Gaussian distribution as a linear regression with latitude as the only independent variable. Haematocrit was only measured at a single temperature (31.5°C). No random factor was included within the haematocrit model.

When modelling enzyme activity for LDH, CS, and the ratio of LDH:CS, a Gaussian distribution with an interaction between latitude and temperature, as well as sample tissue mass (centred) as a co-variate, was used. Additionally, citrate synthase was modelled with a log-link function. Temperature was modelled as a continuous second order polynomial for LDH and CS, and linearly for LDH:CS.

### Software

All statistical analysis was conducted in R (v 4.2.2; R Core Team, 2022). During the model selection analysis GLMs were run using the ‘glm’ function via the ‘*stats*’ (v.4.2.2; R Core Team, 2022) package. GLMMs were run using the ‘glmmTMB’ function within the ‘*glmmTMB*’ package (v.1.1.5; Brooks *et al.*, 2017). Model selection occurred using the function ‘AICc’ and ‘r.squaredGLMM’ (or ‘r.squaredLR’) via the ‘*MuMIn*’ (v.1.47.1; Bartoń, 2023), and ‘BIC’ from ‘*stats*’. Visual and statistical performance of models was checked via both the ‘check_model’ function in the ‘*performance*’ (v. 0.10.0; Ludecke *et al.*, 2021) package and the ‘simulateRedisuals’ and ‘testResiduals’ functions in the ‘*DHARMa*’ (v. 0.4.6; Hartig, 2022) package. The function ‘Anova’ from the ‘*car*’ (Fox and Weisburg, 2019; v3.1–2) package was used to determine the statistical significance of independent variables and interactions. The ‘*emmeans*’ (v. 1.8.2; Lenth, 2023) package was used to extract estimated marginal trends and means from models that were used to test for statistical significance, as well as 95% confidence intervals, and calculated effect sizes (Cohen’s *d*). All figures were made using the ‘*ggplot2*’ (v. 3.4.0; Wickham, 2016) package. Trend lines within figures were created via estimated marginal means (predicted values) for response variables using the ‘emmeans’ functions from the ‘*emmeans*’ package (Lenth, 2023). Data on figures were determined via fitted values (fitted values = predicted values—residuals), where predicted values and residuals were determined by the ‘predict’ and ‘residuals’ function, respectively, from the ‘*stats*’ (R Core Team, 2022) package.

## Results

### Aerobic physiology

**Figure 2:**
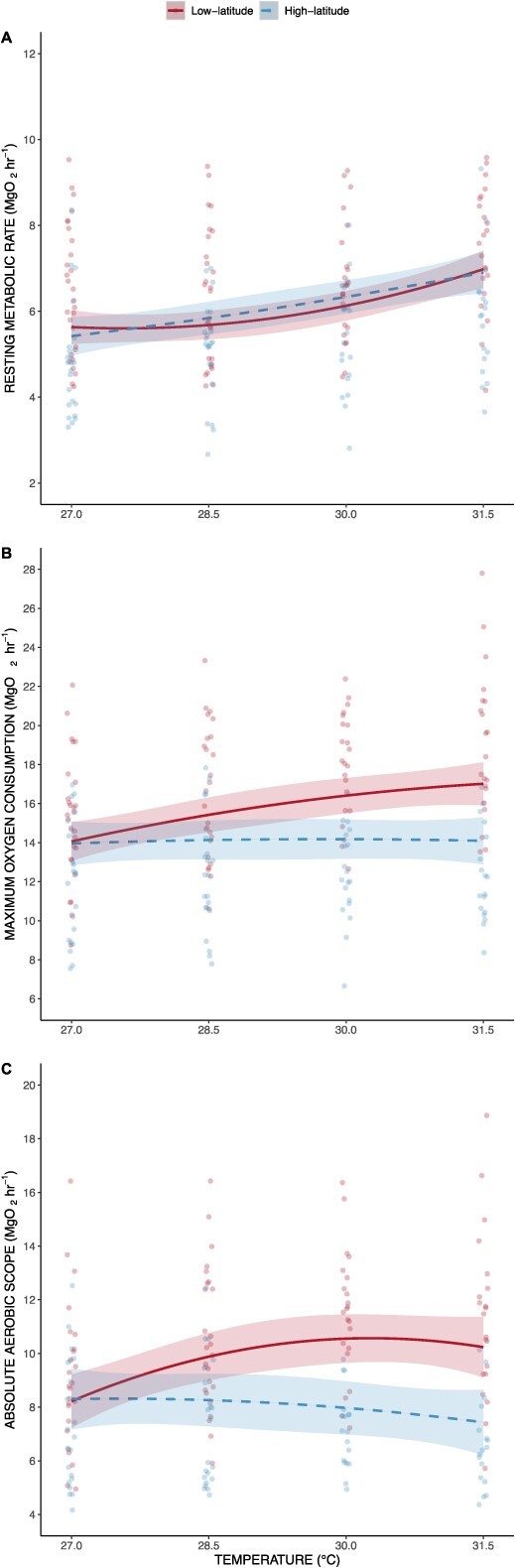
Thermal performance curves of A) resting oxygen performance, B) maximum oxygen performance and C) absolute aerobic scope of fish from low- (solid lines) and high-latitudinal (dashed line) regions across four different temperatures (i.e. 27, 28.5, 30 and 31.5°C). Ribbon represents 95% confidence intervals.

MO_2rest_ displayed a positive relationship with temperature (χ^2^ = 51.57, df = 2, *P* < 0.001), but no significant differences were seen between regions (χ^2^ = 0.003, df = 1, *P* = 0.95) and when comparing the thermal performance curve of fish from low- and high-latitude regions (χ^2^ = 1.82, df = 2, *P* = 0.40; Fig. 2a). The largest increase in MO_2rest_ (14%) between temperature intervals within high-latitude region fish was observed between 28.5 and 30°C. The largest increase in MO_2rest_ (14%) with low-latitude region fish was observed between 30 and 31.5°C.

A positive relationship was seen between MO_2max_ and temperature (χ^2^ = 16.28, df = 2, *P* < 0.001), but the thermal sensitivity of MO_2max_ displayed diverging patterns between low- and high-latitude regions (χ^2^ = 11.18, df = 2, *P* < 0.01). Low-latitude fish had a significantly higher thermal performance curve for MO_2max_ compared to high-latitude region fish (t-ratio = 3.34, *P* = 0.0010, CI [0.26–1.02], *d* = 0.83; Fig. 2b). MO_2max_ of fish from the low-latitude region displayed a plateauing relationship with temperature, increasing by 11% (27–28.5°C), 7% (28.5–30°C) and 4% (30–31.5°C) between measured temperature intervals. Contrastingly, fish from the high-latitude region had a relatively flat response to temperature; MO_2max_ values were constantly ~14.1 mg O_2_ h^−1^. In fish from the low-latitude region the biggest divergence in MO_2max_ values was observed at 30 and 31.5°C, where values were 15% (+2.21 mg O_2_ h^−1^) and 21% (+2.92 mg O_2_ h^−1^) higher than fish from the high-latitude region, respectively.

AAS was positively influenced by temperature (χ^2^ = 6.52, df = 2, *P* = 0.038), and displayed a significant difference between latitudinal regions (χ^2^ = 6.54, df = 1, *P* = 0.011), as well as a significant interaction between temperature and latitudinal region (χ^2^ = 11.46, df = 2, *P* = 0.0032). Low-latitude fish had a higher thermal performance curve compared to high-latitude fish (t-ratio = 3.34, *P* = 0.0010, CI [0.28–1.10], *d* = 0.94; Fig. 2c). AAS was similar between regions at 27°C (~8.35 mg O_2_ h^−1^); however, values diverged at warmer temperatures. At warmer temperatures, 30 and 31.5°C, low-latitude fish exhibited 33% greater (+2.64 mg O_2_ h^−1^) and 38% greater (+2.79 mg O_2_ h^−1^) AAS, respectively, compared to high-latitude fish.

### Immune response

**Figure 3:**
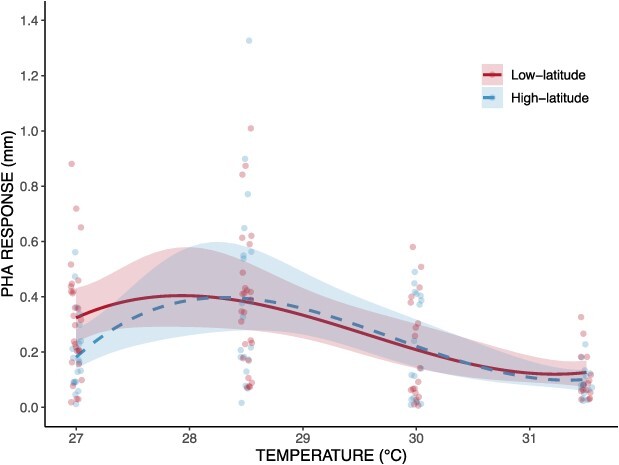
Thermal performance curve of immune swelling response of the caudal peduncle ~18–24 h post-injection of phytohaemagglutinin across four experimental temperatures (i.e. 27, 28.5, 30 and 31.5°C). Solid lines represent low-latitude populations. Dashed line represents high-latitude populations. Ribbon represents 95% confidence intervals.

Immune swelling response exhibited a thermal performance curve that was significantly correlated with temperature (χ^2^ = 50.88, df = 3, *P* < 0.001) and peaked at 28.5°C in both low- and high-latitude regions. The mean caudal peduncle width measured across all samples taken was $\overline{\mathrm{\mu}}$ = 4.43 mm; however, this value varied between temperatures as sample sizes were decreased at higher temperatures due to mortality (Supplementary Table S5). At 27, 28.5, 30 and 31.5°C, fish displayed an average change of +0.25 mm (+6.18%), +0.34 (+7.93%), +0.16 (+3.75%) and + 0.055 (+1.28%) when comparing the caudal peduncle width pre- and post-injection. No significant differences in immune response were found when comparing latitudes (χ^2^ = 1.59, df = 1, *P* = 0.21) or when comparing the thermal performance curves of immune response (χ^2^ = 3.04, df = 3, *P* = 0.38; Fig. 3).

### Haematocrit

Whilst there was a trend of slightly higher haematocrit level in high-latitude populations, no significant difference was observed in haematocrit levels between low- and high-latitude populations at 31.5°C (χ^2^ = 3.84, df = 1, *P* = 0.058; Supplementary Fig. S4). Packed red blood cells composed 22.4 and 25.9% of whole blood for low- and high-latitude populations, respectively.

### Enzyme analysis

**Figure 4:**
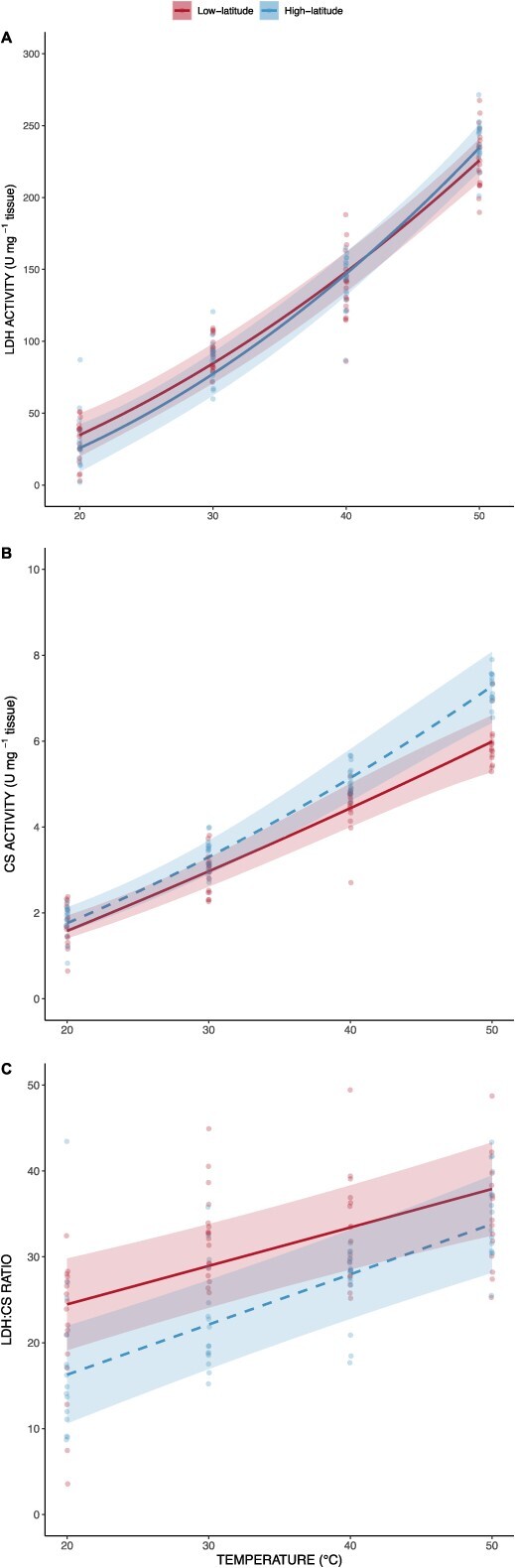
Thermal performance curve of maximal activity of A) LDH, B) CS and C) LDH:CS ratio of low- (solid line) and high-latitudinal (dashed line) populations across four experimental temperatures (i.e. 20, 30, 40 and 50°C). Ribbons represent 95% confidence intervals.

LDH activity was positively correlated with temperature (*χ^2^* = 1862.85, df = 2, *P* < 0.001); however, no significant differences were seen between latitudinal regions (*χ^2^* = 0.05, df = 1, *P* = 0.82) or thermal performance curves of low- and high-latitude fish (χ^2^ = 4.08, df = 2, *P* = 0.13; Fig. 4a). Citrate synthases was also positively correlated with temperature (χ^2^ = 1234.79, df = 2, *P* < 0.001). High-latitude region fish were shown to have significantly higher CS activity level on average than low-latitude region fish (χ^2^ = 4.32, df = 1, *P* = 0.038). Overall, there was no significant difference in the slope of CS thermal performance curves between low- and high-latitude populations (χ^2^ = 3.52, df = 2, *P* = 0.17; Fig. 4b). Low- and high-latitude region CS activity slopes drift apart at 40 and 50°C where CS activity was 1.17 and 1.22 times higher in high-latitude fish compared to low-latitude fish, respectively. However, the lack of significance in CS activity slopes between regions is likely driven by the similarity of results at 20 and 30°C. At 20 and 30°C, CS activity amongst the high-latitude region was just 1.14 and 1.08, respectively, higher than the low-latitude region. Additionally, results at 20 and 30°C showed much smaller absolute values as well as variance. LDH:CS ratio was positively correlated with temperature (χ^2^ = 79.47, df = 1, *P* < 0.001), but no significant difference was observed between latitudes (χ^2^ = 2.96, df = 1, *P* = 0.09) or the slopes of their thermal performance curves (χ^2^ = 1.41, df = 1, *P* = 0.24; Fig. 4c**)**.

## Discussion

How populations respond to climate change will depend on traits that are adapted to localized environmental conditions. Localized environmental conditions can influence thermal preferences and limits within populations via plastic and evolutionary mechanisms, creating a complex adaptive landscape across species’ distributions (Huey *et al.*, 2012; Valladares *et al.*, 2014). Identifying existing intraspecific variation is therefore essential to accurately predict populations’ (and therefore species’) responses to climate change. Our study found evidence of co-gradient variation (i.e. phenotypes align with the observed thermal gradient) within oxygen consumption, absolute aerobic scope, and CS (aerobic) enzyme activity, suggesting that these traits are adapted to localized environmental conditions. However, no intraspecific variation was found in several other traits including immunocompetence, haematocrit, and LDH enzyme activity.

Evidence of co-gradient variation was observed in maximum oxygen consumption and absolute aerobic scope. Fish from the low-latitude region displayed a higher capacity for MO_2max_ and AAS at 30 and 31.5°C, compared to high-latitude conspecifics. Fish from the low-latitude region exhibited rising MO_2max_ and MO_2rest_ with warming; however, high-latitude populations displayed a plateaued MO_2max_ across the testing temperature range and consequently slightly reduced AAS, due to increasing MO_2rest_. Increased absolute aerobic scope at higher temperatures suggests fish from the low-latitude region are currently better equipped to respond to warmer temperatures (within generation), compared to high-latitude conspecifics. According to the Oxygen and Capacity Limitation of Thermal Tolerance (OCLTT; Pörtner and Knust, 2007), AAS can serve as a proxy for the limits of oxygen-demanding processes (e.g. motor activity, reproductive output, growth) that can be performed simultaneously and is expected to be a primary mechanism that determines how fish will respond to climate change (Pörtner *et al.*, 2017). However, the OCLTT hypothesis remains contested within the literature (Clark *et al.*, 2013; Lefevre *et al.*, 2021). To further understand how results from this study related to fitness and performance under projected future conditions, additional studies should aim to link interpopulation differences in MO_2max_ and AAS with reproductive metrics, as well as consider the potential to rapidly shift phenotypes via plasticity.

Despite differences in AAS between regions there was no significant difference in haematocrit. Stronger selection pressures on alternative traits, balancing selection, and/or physiochemical limitations may provide a mechanism for why no significant differences were identified between low- and high-latitude regions for these traits. It is common to observe varying thermal responses between tested traits within experiments. For example, when examining thermal regulation behaviour in high- and low-elevation populations of the jacky lizard (*Amphibolurus muricatus*), Hodgson and Schwanz (2019) found significant differences in panting behaviour, but not basking intensity or duration. Immune response within several species, including *Drosophilia melanogaster* (Early and Clark, 2017) and three-spined stickleback (*Gasterosteus aculeatus*; Robertson *et al.*, 2017), has been characterized by purifying selection and a lack of local adaptation. Considering the observed pattern in AAS, we might have expected latitudinal differences in haematocrit (a proxy for oxygen-carrying capacity of circulatory system; Gallaugher *et al.*, 1995). In the case of the coral reef snapper (*Lutjanus carponotatus*), exposure to a marine heatwave of 29.5 and 30.5°C (+1–2°C) for 4 weeks resulted in an increase in haematocrit to allow maintenance of aerobic capacity (McMahon *et al.*, in review). However, haematocrit was shown to be unresponsive in both the fusilier *Caesion cuning* and the cardinalfish *Cheilodipterus quinquelineatus* when exposed to elevated temperatures (+3.0°C above ambient temperature) for 5 weeks (Johansen *et al.*, 2021).

CS activity was significantly higher in the high-latitude populations compared to the low-latitude regions and may represent a potential adaptation to cooler environmental conditions. As temperatures shift away from thermal optima, towards cooler temperatures, the ability to produce ATP can be inhibited via decreased rates of chemical reactions, rates of diffusion and membrane fluidity (Lucassen *et al.*, 2006; Dhillon and Schulte, 2011). To offset ATP deficiency in cooler environments conspecifics have increased mitochondrial volume density compared to warmer latitude conspecifics (Guderley, 2004). This co-gradient pattern has been observed across closely related species (Johnston *et al.*, 1998, various Perciformes), as well as conspecifics from cold and warm environments (Lucassen *et al.*, 2006; *Gadus morhua*). The pattern of overall higher CS (proxy for mitochondrial volume density) activity in high-latitude populations compared to low-latitude populations suggests that enzymatic maximum respiratory capacity is an unlikely candidate for directly causing whole-organism failure. Growing evidence suggests that whole-organism failure is not driven by a single mechanism, but rather can occur through multiple different mechanisms that can vary between species and contexts (Ern *et al.*, 2023). However, common patterns have emerged suggesting major mechanisms such as ATP synthetic capacity, proton leakage, and the buildup of reactive oxygen species (i.e. indirect results of aerobic respiration) may be more relevant at upper thermal limits (Chung and Schulte, 2020; Ern *et al.*, 2023), particularly within cardiac tissue that has a clear functional link with whole-organism performance (Farrell, 2009; Eliason *et al.*, 2011; Ekström *et al.*, 2017; Nyboer and Chapman, 2018; Pichaud *et al.*, 2019). However, testing upper thermal tolerance mechanisms within cardiac muscle of coral reef fish remains challenging due to size of tissue available for sampling.

No significant differences were identified in the thermal performance curves of LDH or LDH:CS when comparing fish from low- and high-latitude regions. LDH and CS are proxy representations for anaerobic glycolysis and aerobic capacity that be can achieved via the citric acid cycle, respectively (Dahlhoff, 2004; Savoie *et al.*, 2008; Ekström *et al.*, 2017; Pichaud *et al.*, 2019). The positive relationship between temperature and LDH:CS ratios indicate that as temperatures warm there is a greater reliance on anaerobic metabolism, a pattern that is expected amongst ectotherms, and which has previously been identified in several taxa including, crown-of-thorns sea stars (Lang *et al.*, 2021; *Acanthaster spp.*), dogfish sharks (Bouyoucos *et al.*, 2023; *Squalus suckleyi*), Antarctic notothenioids (Mark *et al.*, 2002; Jayasundara *et al.*, 2013) and coral reef fish (Illing *et al.*, 2020; Johansen *et al.*, 2021). However, increased reliance on anaerobic metabolism can become unstable over long periods of time, due to the availability of finite fermentable substrates and cytotoxicity (Rosa *et al.*, 2016).

Whilst there was no latitudinal difference in immunocompetence, there was a negative relationship with temperature. If the reduced swelling at elevated temperatures was due to a lack of available energy, *A. polyacanthus* may be immunocompromised prior to impacts on aerobic capacity, especially in the low-latitude region. A similar response has been observed in another coral reef fish from a similar latitude, the rabbitfish *Siganus doliatus*, where immunocompetence was negligible at 31.5°C (LaMonica *et al.*, 2021). Whilst immunological research in fish is emerging and scarce compared to other taxa, within bird species PHA swelling responses have been shown to be less costly than other activities (e.g. moulting, breeding; Martin *et al.*, 2006). If similar conditions exist within fish, we expect energetically demanding behaviours, such as reproduction, to be reduced or cease prior to declines in absolute aerobic scope. Evidence of such trade-offs have been previously demonstrated in *A. polyacanthus* where reproductive output (i.e. clutch size × egg area) was reduced at temperatures >28.5°C when fish were placed on a high-food diet and ceased on a low-food diet (Donelson *et al.*, 2010). Additionally, when *A. polyacanthus* were acclimated to +3.0°C for two generations, restoration of aerobic capacity was observed, but not reproduction (Donelson *et al.*, 2016; Bernal *et al.*, 2018) Collectively, results provide support for the multiple performance–multiple optima hypothesis (Clark *et al.*, 2013) and highlights the need to study a range of performance fitness-related metrics. There is the potential that repeated PHA injections may allow for acquired immune response as previous research in blue-footed boobies (*Sula nebouxii*) detected an average increase of 90% between first and second PHA injections; attributing the increase to acquired T-mediated immunity (Santiago-Quesada *et al.*, 2015). Thus, the increased swelling at 28.5°C compared to 27°C we observed may be indicating an acquired immune response. Results from this study provide initial evidence of reduced immunocompetence at warmer temperatures; however, this should be interpreted cautiously as an understanding of how repeated PHA measures affect acquired immune response mechanisms remains unclear.

Evidence of co-gradient variation in aerobic capacity suggests that for the populations examined, phenotype and environmental influences are aligned. However, countergradient variation between *A. polyacanthus* populations was previously identified when comparing low-latitude (i.e. Lizard Island) and high-latitude (i.e. Heron Island) populations, which are further north and south than the populations examined in this study, respectively (Gardiner *et al.*, 2010). Gardiner *et al.* (2010) sampled juvenile fish from shallow lagoons, whereas fish in this study were adults collected from ~7 to 12 m on coral reef slope. Reef flats and lagoons generally experience greater thermal variability (minimums, maximums and magnitude of diurnal variation) via exposure to semidiurnal tidal oscillations compared to reef slopes that are exposed to the open ocean and hence more thermally stable, and this is true for the lagoon sites at Heron Island (Brown *et al.*, 2023). Additionally, *A. polyacanthus* from Heron Island have been shown to have high capacity for phenotypic plasticity (Donelson and Munday, 2012; Ryu *et al.*, 2018). In this study, evidence suggests that co-gradient thermal variation can be seen across populations; however, previous findings (Gardiner *et al.*, 2010; Donelson and Munday, 2012) suggest a caveat that biogeographic differences (e.g. water depth, thermal variability) at local scales can disrupt larger biogeographical patterns.

Determining spatial patterns of thermal adaptation underpins the ability to predict population responses to climate change (Sorte *et al.*, 2011; Moran *et al.*, 2016). Climate envelope models frequently assign populations identical thermal tolerances; however, such approaches risk inaccurately projecting species trajectories by ignoring intrapopulation variation. Findings from this experiment demonstrated different oxygen consumption capacity amongst *A. polyacanthus* populations from low- and high-latitude regions as well as a decline in immune response and increased reliance on anaerobic pathways within both regions as temperatures warm. Evidence from previous research further suggests that predicting species responses to climate change would additionally benefit from considering the ability for populations to respond via plastic responses (Donelson *et al.*, 2012), as well as differences in plastic potential between populations (Donelson and Munday, 2012). Furthermore, when results from this study are examined concurrently with Gardiner *et al.* (2010), it suggests that fine-scale biogeographic features can create pockets of adaptive heterogeneity, and that thermal tolerance may be tied closer to mean summer ranges rather than mean summer averages. These findings indicate that the adaptive landscape of species within marine environments may resemble a heterogenous mixture of populations with varying levels of adaptability. Therefore, it is necessary to sample populations from different environments to understand species’ adaptive landscape.

## Supplementary Material

Web_Material_coae058

## Data Availability

The data underlying this article are available on GitHub, at https://github.com/schmidte10/CONPHYS-2024

## References

[ref1] Aitken SN , Bemmels JB (2016) Time to get moving: assisted gene flow of forest trees. Evol Appl 9(1): 271–290 10.1111/eva.12293. 27087852 PMC4780373

[ref2] Aitken SN , Whitlock MC (2013) Assisted gene flow to facilitate local adaptation to climate change. Annu Rev Ecol Evol Syst 44(1): 367–388 10.1146/annurev-ecolsys-110512-135747.

[ref3] Allen GR (1991) Damselfishes of the World. Mergus Publishers, Melle, Germany

[ref4] Angilletta MJ , Oufiero CE, Sears MW (2004) Thermal adaptation of maternal and embryonic phenotypes in a geographically widespread ectotherm. Int Congr Ser 1275: 258–266. 10.1016/j.ics.2004.07.038.

[ref5] Australian Institute of Marine Science (AIMS) (2020) AIMS Sea Water Temperature Observing System (AIMS Temperature Logger Program). https://apps.aims.gov.au/ts-explorer/ (date last accessed 1 October 2020)

[ref6] Bartoń K (2023) MuMIn: multi-model inference. R package version 1.47.5. http://CRAN.R-project.org/package=MuMIn (date last accessed 12 June 2024).

[ref7] Barton M , Sunnucks P, Norgate M, Murray N, Kearney M (2014) Co-gradient variation in growth rate and development time of a broadly distributed butterfly. PloS One 9(4): 1–8 10.1371/journal.pone.0095258. PMC3990641 24743771

[ref8] Bazzicalupo E , Ratkiewicz M, Seryodkin IV, Okhlopkov I, Galsandorj N, Yarovenko YA, Ozolins J, Saveljev AP, Melovski D, Gavashelishvili A et al. (2023) Environment association analyses reveal geographically restricted adaptive divergence across the range of the widespread Eurasian carnivore *Lynx lynx* (Linnaeus, 1758). Evol Appl 00: 1–16. 10.1111/eva.13570. PMC10681490 38029067

[ref9] Bennett S , Duarte CM, Marbà N, Wernberg T (2019) Integrating within-species variation in thermal physiology into climate change ecology. Philos Trans R Soc B Biol Sci 374(1778. 20180550. 10.1098/rstb.2018.0550 PMC6606463 31203756

[ref10] Bernal MA , Donelson JM, Veilleux HD, Ryu T, Munday PL, Ravasi T (2018) Phenotypic and molecular consequences of stepwise temperature increase across generations in a coral reef fish. Mol Ecol 27(22): 4516–4528 10.1111/mec.14884. 30267545

[ref123] Blank JM , Morrissette JM, Landeira-Fernandez AM, Blackwell SB, Williams TD, Block BA (2004) In situcardiac performance of Pacific bluefin tuna hearts in response to acute temperature change. Journal of Experimental Biology, 207(5): 881–890. 10.1242/jeb.00820. 14747418

[ref124] Bleeker P (1855) Derde bijdrage tot de kennis der ichthyologische fauna van Batjan. Natuurkundig Tijdschrift voor Nederlandsch Indië 93:491–504.

[ref11] Bouyoucos IA , Weinrauch AM, Jeffries KM, Anderson WG (2023) Physiological responses to acute warming at the agitation temperature in a temperate shark. J Exp Biol 226(19): 1–14. 10.1242/JEB.246304/329029/AM/PHYSIOLOGICAL-RESPONSES-TO-ACUTE-WARMING-AT-THE. 37721037

[ref12] Brooks ME , Kristensen K, van Benthem KJ, Magnusson A, Berg CW, Nielsen A, Skaug HJ, Maechler M, Bolker BM (2017) Glmm TMB balances speed and flexibility among packages for zero-inflated generalized linear mixed modeling. R J 9(2): 378–400 10.32614/RJ-2017-066

[ref13] Brown KT , Eyal G, Dove SG, Barott KL (2023) Fine-scale heterogeneity reveals disproportionate thermal stress and coral mortality in thermally variable reef habitats during a marine heatwave. Coral Reefs 42(1): 131–142 10.1007/s00338-022-02328-6. 36415309 PMC9672654

[ref14] Calosi P , Bilton DT, Spicer JI (2008) Thermal tolerance, acclimatory capacity and vulnerability to global climate change. Biol Lett 4(1): 99–102 10.1098/rsbl.2007.0408. 17986429 PMC2412917

[ref15] Chabot D , Steffensen JF, Farrell AP (2016) The determination of standard metabolic rate in fishes. J Fish Biol 88(1): 81–121 10.1111/jfb.12845. 26768973

[ref16] Chiono A , Paul JR (2023) The climatic variability hypothesis and trade-offs in thermal performance in coastal and inland populations of Mimulus guttatus. Evolution 77(3): 870–880 10.1093/evolut/qpad005. 36637137

[ref17] Chung DJ , Schulte PM (2020) Mitochondria and the thermal limits of ectotherms. J Exp Biol 223(3): 1–10 10.1093/evolut/qpad005. PMC10668358 33109621

[ref18] Clark TD , Sandblom E, Jutfelt F (2013) Aerobic scope measurements of fishes in an era of climate change: respirometry, relevance and recommendations. J Exp Biol 216(15): 2771–2782 10.1242/jeb.084251. 23842625

[ref19] Conover D , Schultz ET (1995) Significance of countergradient variation. Trends Ecol Evol 10(6): 248–252 10.1016/S0169-5347(00)89081-3. 21237029

[ref20] Conover DO , Duffy TA, Hice LA (2009) The covariance between genetic and environmental influences across ecological gradients: reassessing the evolutionary significance of countergradient and cogradient variation. Ann N Y Acad Sci 1168(1): 100–129 10.1111/j.1749-6632.2009.04575.x. 19566705

[ref21] Dahlhoff EP (2004) Biochemical indicators of stress and metabolism: applications for marine ecological studies. Annu Rev Physiol 66(1): 183–207 10.1146/annurev.physiol.66.032102.114509. 14977401

[ref22] Des Roches S , Post DM, Turley NE, Bailey JK, Hendry AP, Kinnison MT, Schweitzer JA, Palkovacs EP (2018) The ecological importance of intraspecific variation. Nat Ecol Evol 2(1): 57–64 10.1038/s41559-017-0402-5. 29203921

[ref23] Deutsch CA , Tewksbury JJ, Huey RB, Sheldon KS, Ghalambor CK, Haak DC, Martin PR (2008) Impacts of climate warming on terrestrial ectotherms across latitude. PNAS 105(18): 6668–6672 10.1073/pnas.0709472105. 18458348 PMC2373333

[ref24] Dhillon RS , Schulte PM (2011) Intraspecific variation in the thermal plasticity of mitochondria in killifish. J Exp Biol 214(21): 3639–3648 10.1242/jeb.057737. 21993793

[ref25] Doherty PJ , Mather P, Planes S (1994) Acanthochromis polyacanthus, a fish lacking larval dispersal, has genetically differentiated populations at local and regional scales on the Great Barrier Reef. Mar Biol 121(1): 11–21 10.1007/BF00349469.

[ref26] Donelson JM , Munday PL (2012) Thermal sensitivity does not determine acclimation capacity for a tropical reef fish. J Anim Ecol 81(5): 1126–1131 10.1111/j.1365-2656.2012.01982.x. 22433064

[ref27] Donelson JM , Munday PL, McCormick MI, Pankhurst NW, Pankhurst PM (2010) Effects of elevated water temperature and food availability on the reproductive performance of a coral reef fish. Mar Ecol Prog Ser 401: 233–243. 10.3354/meps08366.

[ref28] Donelson JM , Munday PL, McCormick MI, Pitcher CR (2012) Rapid transgenerational acclimation of a tropical reef fish to climate change. Nat Clim Chang 2(1): 30–32 10.1038/nclimate1323.

[ref29] Donelson JM , Sunday JM, Figueira WF, Gaitán-Espitia JD, Hobday AJ, Johnson CR, Leis JM, Ling SD, Marshall D, Pandolfi JM, et al. (2019) Understanding interactions between plasticity, adaptation and range shifts in response to marine environmental change. Philos Trans R Soc B Biol Sci 374(1768): 20180186 10.1098/rstb.2018.0186. PMC6365866 30966966

[ref30] Donelson JM , Wong M, Booth DJ, Munday PL (2016) Transgenerational plasticity of reproduction depends on rate of warming across generations. Evol Appl 9(9): 1072–1081 10.1111/eva.12386. 27695516 PMC5039321

[ref31] Donelson JM , Yasutake YC (2024) Pilot study to determine phytohaemagglutinin assay as a proxy for immune response in Acanthochromis polyacanthus. 10.25903/2hmd-8629 (data last accessed 1 January 2019).

[ref32] Early AM , Clark AG (2017) Genomic signatures of local adaptation in the Drosophila immune response. Fly (Austin) 11(4): 277–283 10.1080/19336934.2017.1337612. 28586288 PMC5721942

[ref33] Ekström A , Sandblom E, Blier PU, Cyr BAD, Brijs J, Pichaud N (2017) Thermal sensitivity and phenotypic plasticity of cardiac mitochondrial metabolism in European perch, *Perca fluviatilis*. J Exp Biol 220(Pt 3): 386–396 10.1242/jeb.150698. 27852753

[ref34] Eliason EJ , Clark TD, Hague MJ, Hanson LM, Gallagher ZS, Jeffries KM, Gale MK, Patterson DA, Hinch SG, Farrell AP (2011) Differences in thermal tolerance among sockeye salmon populations. Science 332(6025): 109–112 10.1126/science.1199158. 21454790

[ref35] Ern R , Andreassen AH, Jutfelt F (2023) Physiological mechanisms of acute upper thermal tolerance in fish. Phys Ther 38(3): 141–158 10.1152/physiol.00027.2022. 36787401

[ref36] Farrell AP (2009) Environment, antecedents and climate change: lessons from the study of temperature physiology and river migration of salmonids. J Exp Biol 212(23): 3771–3780 10.1242/jeb.023671. 19915118

[ref37] Fox J , Weisburg S (2019) An R Companion to Applied Regression. 3rd edn, Sage, Thousand Oaks, CA. https://socialsciences.mcmaster.ca/jfox/Books/Companion/index.html.

[ref38] Gallaugher P , Thorarensen H, Farrell AP (1995) Hematocrit in oxygen transport and swimming in rainbow trout (*Oncorhynchus mykiss*). Respir Physiol 102(2–3): 279–292 10.1016/0034-5687(95)00065-8. 8904019

[ref39] Gardiner NM , Munday PL, Nilsson GE (2010) Counter-gradient variation in respiratory performance of coral reef fishes at elevated temperatures. PloS One 5(10): e13299 10.1371/journal.pone.0013299. 20949020 PMC2952621

[ref40] Guderley H (2004) Metabolic responses to low temperature in fish muscle. Biol Rev 79(2): 409–427 10.1017/S1464793103006328. 15191230

[ref41] Gunderson AR , Stillman JH (2015) Plasticity in thermal tolerance has limited potential to buffer ectotherms from global warming. Proc R Soc B Biol Sci 282(1808 20150401. 10.1098/rspb.2015.0401 PMC4455808 25994676

[ref42] Harianto J , Carey N, Byrne M (2019) respR - an R package for the manipulation and analysis of respirometry data. Methods Ecol Evol 10(6): 912–920 10.1111/2041-210X.13162.

[ref43] Hartig F (2022) DHARMa: residual diagnostics for hierarchical (multi-level / mixed) regression models. R package version 0.4.6. http://CRAN.R-project.org/package=DHARMa (data last accessed 12 June 2024).

[ref44] Hodgson MJ , Schwanz LE (2019) Drop it like it’s hot: interpopulation variation in thermal phenotypes shows counter-gradient pattern. J Therm Biol 83: 178–186. 10.1016/j.jtherbio.2019.05.016. 31331517

[ref45] Hoffmann AA , Sørensen JG, Loeschcke V (2003) Adaptation of Drosophila to temperature extremes: bringing together quantitative and molecular approaches. J Therm Biol 28(3): 175–216 10.1016/S0306-4565(02)00057-8.

[ref46] Huey RB , Kearney MR, Krockenberger A, Holtum JAM, Jess M, Williams SE (2012) Predicting organismal vulnerability to climate warming: roles of behaviour, physiology and adaptation. Philos Trans R Soc B Biol Sci 367(1596): 1665–1679 10.1098/rstb.2012.0005. PMC3350654 22566674

[ref47] Illing B , Downie AT, Beghin M, Rummer JL (2020) Critical thermal maxima of early life stages of three tropical fishes: effects of rearing temperature and experimental heating rate. J Therm Biol 90: 102582. 10.1016/j.jtherbio.2020.102582. 32479385

[ref48] IPCC (2021) Summary for policymakers. In V Masson-Delmotte, P Zhai, A Pirani, S Connors, C Péan, S Berger, N Caud, Y Chen, L Goldfarb, M Gomis et al., eds, Climate Change 2021: The Physical Science Basis. Contribution of Working Group I to the Sixth Assessment Report of the Intergovernmental Panel on Climate Change. Cambridge University Press, Cambridge, UK and New York, NY, USA, pp. 3–32

[ref49] Janzen DH (1967) Why mountain passes are higher in the tropics. Am Nat 101(919): 233–249 10.1086/282487.

[ref50] Jayasundara N , Healy TM, Somero GN (2013) Effects of temperature acclimation on cardiorespiratory performance of the Antarctic notothenioid *Trematomus bernacchii*. Polar Biol 36(7): 1047–1057 10.1007/s00300-013-1327-3.

[ref51] Johansen JL , Nadler LE, Habary A, Bowden AJ, Rummer J (2021) Thermal acclimation of tropical coral reef fishes to global heat waves. Elife 10(7): e59162 10.1007/s00300-013-1327-3. 33496262 PMC7837695

[ref52] Johnston IA , Calvo J, Guderley H, Fernandez D, Palmer L (1998) Latitudinal variation in the abundance and oxidative capacities of muscle mitochondria in perciform fishes. J Exp Biol 201(1): 1–12 10.1242/jeb.201.1.1. 9390931

[ref53] Jones GP , Milicich MJ, Emslie MJ, Lunow C (1999) Self-recruitment in a coral reef fish population. Environ Prot 402(6763): 802–804 10.1038/45538.

[ref54] Kelly MW , Griffiths JS (2021) Selection experiments in the sea: what can experimental evolution tell us about how marine life will respond to climate change? Biol Bull 241: 30–42. 10.1086/715109. 34436966

[ref55] Kelly MW , Sanford E, Grosberg RK (2012) Limited potential for adaptation to climate change in a broadly distributed marine crustacean. Proc R Soc B 279(1727 349–356 10.1098/rspb.2011.0542. PMC3223665 21653591

[ref56] Kuo ESL , Sanford E (2009) Geographic variation in the upper thermal limits of an intertidal snail: implications for climate envelope models. Mar Ecol Prog Ser 388: 137–146. 10.3354/meps08102.

[ref57] LaMonica LE , Fox RJ, Donelson JM (2021) Thermal sensitivity of juvenile rabbitfishes *Siganus doliatus* and *S. lineatus* (Siganidae): a key role for habitat? Coral Reefs 40(4): 1307–1320 10.1007/s00338-021-02146-2.

[ref58] Lang BJ , Donelson JM, Caballes CF, Doll PC, Pratchett MS (2021) Metabolic responses of Pacific crown-of-thorns sea stars (Acanthaster sp.) to acute warming. Biol Bull 241(3): 347–358 10.1086/717049. 35015619

[ref59] Lefevre S , Wang T, McKenzie DJ (2021) The role of mechanistic physiology in investigating impacts of global warming on fishes. J Exp Biol 224: 1–13. 10.1242/jeb.238840 33627469

[ref60] Lenoir J , Bertrand R, Comte L, Bourgeaud L, Hattab T, Murienne J, Grenouillet G (2020) Species better track climate warming in the oceans than on land. Nat Ecol Evol 4(8): 1044–1059 10.1038/s41559-020-1198-2. 32451428

[ref61] Lenth R (2023) emmeans: estimated marginal means, aka least-square means. R package version 1.8.8. http://CRAN.R-project.org/package=emmeans (date last accessed 12 June 2024).

[ref62] Liepe KJ , Hamann A, Smets P, Fitzpatrick CR, Aitken SN (2016) Adaptation of lodgepole pine and interior spruce to climate: implications for reforestation in a warming world. Evol Appl 9(2): 409–419 10.1111/eva.12345. 26834833 PMC4721073

[ref63] Lucassen M , Koschnick N, Eckerle LG, Pörtner HO (2006) Mitochondrial mechanisms of cold adaptation in cod (Gadus morhua L.) populations from different climatic zones. J Exp Biol 209(13): 2462–2471 10.1242/jeb.02268. 16788029

[ref64] Ludecke D , Ben-Shachar MS, Patil I, Waggoner P, Makowski D (2021) Performance: an R package for assessment, comparison and testing of statistical models. J Open Source Softw 6(60): 3139 10.21105/joss.03139.

[ref65] Ludt WB , Rocha LA (2015) Shifting seas: the impacts of Pleistocene sea-level fluctuations on the evolution of tropical marine taxa. J Biogeogr 42(1): 25–38 10.1111/jbi.12416.

[ref66] Mahony CR , MacLachlan IR, Lind BM, Yoder JB, Wang T, Aitken SN (2020) Evaluating genomic data for management of local adaptation in a changing climate: a lodgepole pine case study. Evol Appl 13(1): 116–131 10.1111/eva.12871. 31892947 PMC6935591

[ref122] Marcil, J, Swain DP, Hutchings JA (2006) Countergradient variation in body shape between two populations of Atlantic cod (Gadus morhua). Proceedings of the Royal Society B: Biological Sciences, 273(1583): 217–223. 10.1098/rspb.2005.3306. PMC1560025 16555790

[ref67] Mark FC , Bock C, Pörtner HO (2002) Oxygen-limited thermal tolerance in antarctic fish investigated by MRI and 31P-MRS. Am J Physiol - Regul Integr Comp Physiol 283(5 R1254–R1262 10.1152/AJPREGU.00167.2002 12376420

[ref68] Martin LB , Han P, Lewittes J, Kuhlman JR, Klasing KC, Wikelski M (2006) Phytohemagglutinin-induced skin swelling in birds: histological support for a classic immunoecological technique. Funct Ecol 20(2): 290–299 10.1111/j.1365-2435.2006.01094.x.

[ref69] McKenzie DJ , Zhang Y, Eliason EJ, Schulte PM, Claireaux G, Blasco FR, Nati JJH, Farrell AP (2021) Intraspecific variation in tolerance of warming in fishes. J Fish Biol 98: 1536–1555. 10.1111/j.1365-2435.2006.01094.x. 33216368

[ref70] McMahon S , Munday PL, Donelson JM (*In review*) The effects of marine heatwaves on a coral reef snapper: insights into aerobic and anaerobic physiology and recovery potential. In Conserv Physiol, CONPHYS-2023-102.R1 10.1093/conphys/coae060 PMC11793158 39906146

[ref71] Miller-Sims VC , Gerlach G, Kingsford MJ, Atema J (2008) Dispersal in the spiny damselfish, Acanthochromis polyacanthus, a coral reef fish species without a larval pelagic stage. Mol Ecol 17(23): 5036–5048 10.1111/j.1365-294X.2008.03986.x. 19120989

[ref72] Molina-Montenegro MA , Naya DE (2012) Latitudinal patterns in phenotypic plasticity and fitness-related traits: assessing the climatic variability hypothesis (CVH) with an invasive plant species. PloS One 7(10): 23–28 10.1371/journal.pone.0047620. PMC3478289 23110083

[ref73] Moran E V. , Hartig F, Bell DM (2016) Intraspecific trait variation across scales: implications for understanding global change responses. Glob Chang Biol 22(1): 137–150 10.1111/gcb.13000. 26061811

[ref74] Morgan R , Finnøen MH, Jensen H, Pélabon C, Jutfelt F (2020) Low potential for evolutionary rescue from climate change in a tropical fish. PNAS 117(52): 33365–33372 10.1073/pnas.2011419117. 33318195 PMC7776906

[ref75] Naya DE , Spangenberg L, Naya H, Bozinovic F (2012) Latitudinal patterns in rodent metabolic flexibility. Am Nat 179(6. E172–E179 10.1086/665646 22617269

[ref76] Nilsson GE , Crawley N, Lunde IG, Munday PL (2009) Elevated temperature reduces the respiratory scope of coral reef fishes. Glob Chang Biol 15(6): 1405–1412 10.1111/j.1365-2486.2008.01767.x.

[ref77] Norin T , Clark TD (2016) Measurement and relevance of maximum metabolic rate in fishes. J Fish Biol 88(1): 122–151 10.1111/jfb.12796. 26586591

[ref78] Nyboer EA , Chapman LJ (2018) Cardiac plasticity influences aerobic performance and thermal tolerance in a tropical, freshwater fish at elevated temperatures. J Exp Biol 221: 1–14. 10.1242/jeb.178087 29895683

[ref79] Overgaard J , Kristensen TN, Mitchell KA, Hoffmann AA (2011) Thermal tolerance in widespread and tropical Drosophila species: does phenotypic plasticity increase with latitude? Am Nat 178(S1 S80–S96. 10.1086/661780 21956094

[ref80] Pereira RJ , Sasaki MC, Burton RS (2017) Adaptation to a latitudinal thermal gradient within a widespread copepod species: the contributions of genetic divergence and phenotypic plasticity. Proc R Soc B Biol Sci 284(1853): 2017023 10.1098/rspb.2017.0236. PMC5413927 28446698

[ref81] Pichaud N , Ekström A, Breton S, Sundström F, Rowinski P, Blier PU, Sandblom E (2019) Cardiac mitochondrial plasticity and thermal sensitivity in a fish inhabiting an artificially heated ecosystem. Sci Rep 9(1): 1–11 10.1038/s41598-019-54165-3. 31780821 PMC6883045

[ref82] Pinsky ML , Eikeset AM, McCauley DJ, Payne JL, Sunday JM (2019) Greater vulnerability to warming of marine versus terrestrial ectotherms. Nature 569(7754): 108–111 10.1038/s41586-019-1132-4. 31019302

[ref83] Planes S , Doherty PJ, Bernardi G (2001) Strong genetic divergence among populations of a marine fish with limited dispersal, Acanthochromis polyacanthus, within the Great Barrier Reef and the Coral Sea. Evolution (N Y) 55(7754): 2263–2273 10.1038/s41586-019-1132-4. 11794786

[ref84] Plumb WJ , Coker TLR, Stocks JJ, Woodcock P, Quine CP, Nemesio-Gorriz M, Douglas GC, Kelly LJ, Buggs RJA (2020) The viability of a breeding programme for ash in the British Isles in the face of ash dieback. Plants People Planet 2(1): 29–40 10.1002/ppp3.10060.

[ref85] Pörtner HO , Bock C, Mark FC (2017) Oxygen- & capacity-limited thermal tolerance: bridging ecology & physiology. J Exp Biol 220(15): 2685–2696 10.1242/jeb.134585. 28768746

[ref86] Pörtner HO , Knust R (2007) Climate change affects marine fishes through the oxygen limitation of thermal tolerance. Science 315(5808): 95–97 10.1126/science.1135471. 17204649

[ref87] Pratchett MS , Messmer V, Reynolds J, Martin J, Clark TD, Munday PL, Tobin A., Hoey AS (2013) Effects of climate change on reproduction, larval development, and adult health of coral trout (*Plectropomus* Spp.). https://www.fish.gov.au/Archived-Reports/2014/Documents/2014_refs/Pratchett%20et%20al%202013%20FRDC%202010-554.pdf (date last accessed 18 June 2024).

[ref88] R Core Team (2022) R: A Language and Environment for Statistical Computing. R Found Stat Comput Vienna, Austria

[ref89] Robertson DR (1973) Field observations on the reproductive behaviour of a Pomacentrid fish, Acanthochromis polyacanthus. Z Tierpsychol 32(3): 319–324 10.1111/j.1439-0310.1973.tb01108.x. 4781186

[ref90] Robertson S , Bradley JE, MacColl ADC (2017) No evidence of local adaptation of immune responses to Gyrodactylus in three-spined stickleback (*Gasterosteus aculeatus*). Fish Shellfish Immunol 60: 275–281. 10.1016/j.fsi.2016.11.058. 27913248

[ref91] Rosa R , Ricardo Paula J, Sampaio E, Pimentel M, Lopes AR, Baptista M, Guerreiro M, Santos C, Campos D, Almeida-Val VMF, et al. (2016) Neuro-oxidative damage and aerobic potential loss of sharks under elevated CO2 and warming. Mar Biol 163(5): 1–10 10.1007/s00227-016-2898-7.

[ref92] Rummer JL , Couturier CS, Stecyk JAW, Gardiner NM, Kinch JP, Nilsson GE, Munday PL (2014) Life on the edge: thermal optima for aerobic scope of equatorial reef fishes are close to current day temperatures. Glob Chang Biol 20(4): 1055–1066 10.1111/gcb.12455. 24281840 PMC4677772

[ref93] Ryu T , Veilleux HD, Donelson JM, Munday PL, Ravasi T (2018) The epigenetic landscape of transgenerational acclimation to ocean warming. Nat Clim Chang 8(6): 504–509 10.1038/s41558-018-0159-0.

[ref94] Sandblom E , Clark TD, Gräns A, Ekström A, Brijs J, Sundström LF, Odelström A, Adill A, Aho T, Jutfelt F (2016) Physiological constraints to climate warming in fish follow principles of plastic floors and concrete ceilings. Nat Commun 7(1): 1–8 10.1038/ncomms11447. PMC4873662 27186890

[ref95] Sanford E , Kelly MW (2011) Local adaptation in marine invertebrates. Ann Rev Mar Sci 3(1): 509–535 10.1146/annurev-marine-120709-142756. 21329215

[ref96] Santiago-Quesada F , Albano N, Castillo-Guerrero JA, Fernández G, González-Medina E, Sánchez-Guzmán JM (2015) Secondary phytohaemagglutinin (PHA) swelling response is a good indicator of T-cell-mediated immunity in free-living birds. Ibis (Lond 1859) 157(4): 767–773 10.1111/ibi.12295.

[ref97] Sasaki MC , Dam HG (2019) Integrating patterns of thermal tolerance and phenotypic plasticity with population genetics to improve understanding of vulnerability to warming in a widespread copepod. Glob Chang Biol 25(12): 4147–4164 10.1111/gcb.14811. 31449341

[ref98] Savoie A , Le François NR, Cahu C, Blier PU (2008) Metabolic and digestive activity profiles of newly hatched spotted wolffish (*Anarhichas minor Olafsen*): effect of temperature. Aquacult Res 39(4): 382–389 10.1111/j.1365-2109.2007.01797.x.

[ref99] Schmid M , Guillaume F (2017) The role of phenotypic plasticity on population differentiation. Heredity 119(4): 214–225 10.1038/hdy.2017.36. 28745716 PMC5597782

[ref100] Seebacher F , Guderley H, Elsey RM, Trosclair PL (2003) Seasonal acclimatisation of muscle metabolic enzymes in a reptile (*Alligator mississippiensis*). J Exp Biol 206(7): 1193–1200 10.1242/jeb.00223. 12604579

[ref101] Shah AA , Gill BA, Encalada AC, Flecker AS, Funk WC, Guayasamin JM, Kondratieff BC, Poff NLR, Thomas SA, Zamudio KR, et al. (2017) Climate variability predicts thermal limits of aquatic insects across elevation and latitude. Funct Ecol 31(11): 2118–2127 10.1111/1365-2435.12906.

[ref102] Snover ML , Adams MJ, Ashton DT, Bettaso JB, Welsh HH (2015) Evidence of counter-gradient growth in western pond turtles (*Actinemys marmorata*) across thermal gradients. Freshw Biol 60(9): 1944–1963 10.1111/fwb.12623.

[ref103] Somero GN (2010) The physiology of climate change: how potentials for acclimatization and genetic adaptation will determine “winners” and “losers.” J Exp Biol 213(6): 912–920 10.1242/jeb.037473. 20190116

[ref104] Sorte CJB , Jones SJ, Miller LP (2011) Geographic variation in temperature tolerance as an indicator of potential population responses to climate change. J Exp Mar Bio Ecol 400(1–2): 209–217 10.1016/j.jembe.2011.02.009.

[ref105] Stamp MA , Hadfield JD (2020) The relative importance of plasticity versus genetic differentiation in explaining between population differences; a meta-analysis. Ecol Lett 23(10): 1432–1441 10.1111/ele.13565. 32656957

[ref106] Stevens GC (1989) The latitudinal gradient in geographical range: how so many species coexist in the tropics. Am Nat 133(2): 240–256 10.1086/284913.

[ref107] Stillman JH (2002) Causes and consequences of thermal tolerance limits in rocky intertidal porcelain crabs, genus Petrolisthes. Integr Comp Biol 42(4): 790–796 10.1093/icb/42.4.790. 21708777

[ref108] Stillman JH (2003) Acclimation capacity underlies susceptibility to climate change. Science (80-) 301(5629): 65 10.1126/science.1083073. 12843385

[ref109] Stuart-Smith RD , Edgar GJ, Bates AE (2017) Thermal limits to the geographic distributions of shallow-water marine species. Nat Ecol Evol 1(12): 1846–1852 10.1038/s41559-017-0353-x. 29062125

[ref110] Sullivan KM , Somero GN (1980) Enzyme activities of fish skeletal muscle and brain as influenced by depth of occurrence and habits of feeding and locomotion. Mar Biol 60(2–3): 91–99 10.1007/BF00389152.

[ref111] Sunday JM , Bates AE, Dulvy NK (2011) Global analysis of thermal tolerance and latitude in ectotherms. Proc R Soc B Biol Sci 278(1713): 1823–1830 10.1098/rspb.2010.1295. PMC3097822 21106582

[ref112] Swearer SE , Shima JS, Hellberg ME, Thorrold SR, Jones GP, Robertson DR, Morgan SG, Selkoe KA, Ruiz GM, Warner RR (2002) Evidence of self-recruitment in demersal marine populations. Bull Mar Sci 70(1713): 251–271 10.1098/rspb.2010.1295.

[ref113] Taboun ZS , Walter RP, Ovenden JR, Heath DD (2021) Spatial and temporal genetic variation in an exploited reef fish: the effects of exploitation on cohort genetic structure. Evol Appl 145): 1286–1300 10.1111/eva.13198. 34025768 PMC8127707

[ref114] Tewksbury JJ , Huey RB, Deutsch CA (2008) Ecology: putting the heat on tropical animals. Science 320(5881): 1296–1297 10.1126/science.1159328. 18535231

[ref115] Thibault M , Blier PU, Guderley H (1997) Seasonal variation of muscle metabolic organization in rainbow trout (*Oncorhynchus mykiss*). Fish Physiol Biochem 16(2): 139–155 10.1007/BF00004671. 24214209

[ref116] Valladares F , Matesanz S, Guilhaumon F, Araújo MB, Balaguer L, Benito-Garzón M, Cornwell W, Gianoli E, van Kleunen M, Naya DE, et al. (2014) The effects of phenotypic plasticity and local adaptation on forecasts of species range shifts under climate change. Ecol Lett 17(11): 1351–1364 10.1111/ele.12348. 25205436

[ref117] Van Herwerden L , Doherty PJ (2006) Contrasting genetic structures across two hybrid zones of a tropical reef fish, Acanthochromis polyacanthus (Bleeker 1855). J Evol Biol 19(1): 239–252 10.1111/j.1420-9101.2005.00969.x. 16405595

[ref118] Van Herwerden L , Howard Choat J, Newman SJ, Leray M, Hillersøy G (2009) Complex patterns of population structure and recruitment of *Plectropomus leopardus* (Pisces: Epinephelidae) in the Indo-West Pacific: implications for fisheries management. Mar Biol 156(8): 1595–1607 10.1007/s00227-009-1195-0.

[ref119] van Oppen MJH , Puill-Stephan E, Lundgren P, De’ath G, Bay LK (2014) First-generation fitness consequences of interpopulational hybridisation in a Great Barrier Reef coral and its implications for assisted migration management. Coral Reefs 33(3): 607–611 10.1007/s00338-014-1145-2.

[ref120] Wickham H (2016) ggplot2: Elegant Graphics For Data Analysis. Springer-Verlag, New York. http://ggplot2.tidyverse.org (data last accessed 12 June 2024).

[ref121] Yampolsky LY , Schaer TMM, Ebert D (2014) Adaptive phenotypic plasticity and local adaptation for temperature tolerance in freshwater zooplankton. Proc R Soc B Biol Sci 281(1776): 20132744 10.1098/rspb.2013.2744. PMC3871322 24352948

