# Supplementary material for: Regional thermal variation in a coral reef fish
Source: Conserv Physiol. 2024 Aug 13;12(1):coae058. doi: 10.1093/conphys/coae058 (PMC11320370; doi:10.1093/conphys/coae058)
Supplement: Web_Material_coae058 [file web_material_coae058.pdf]

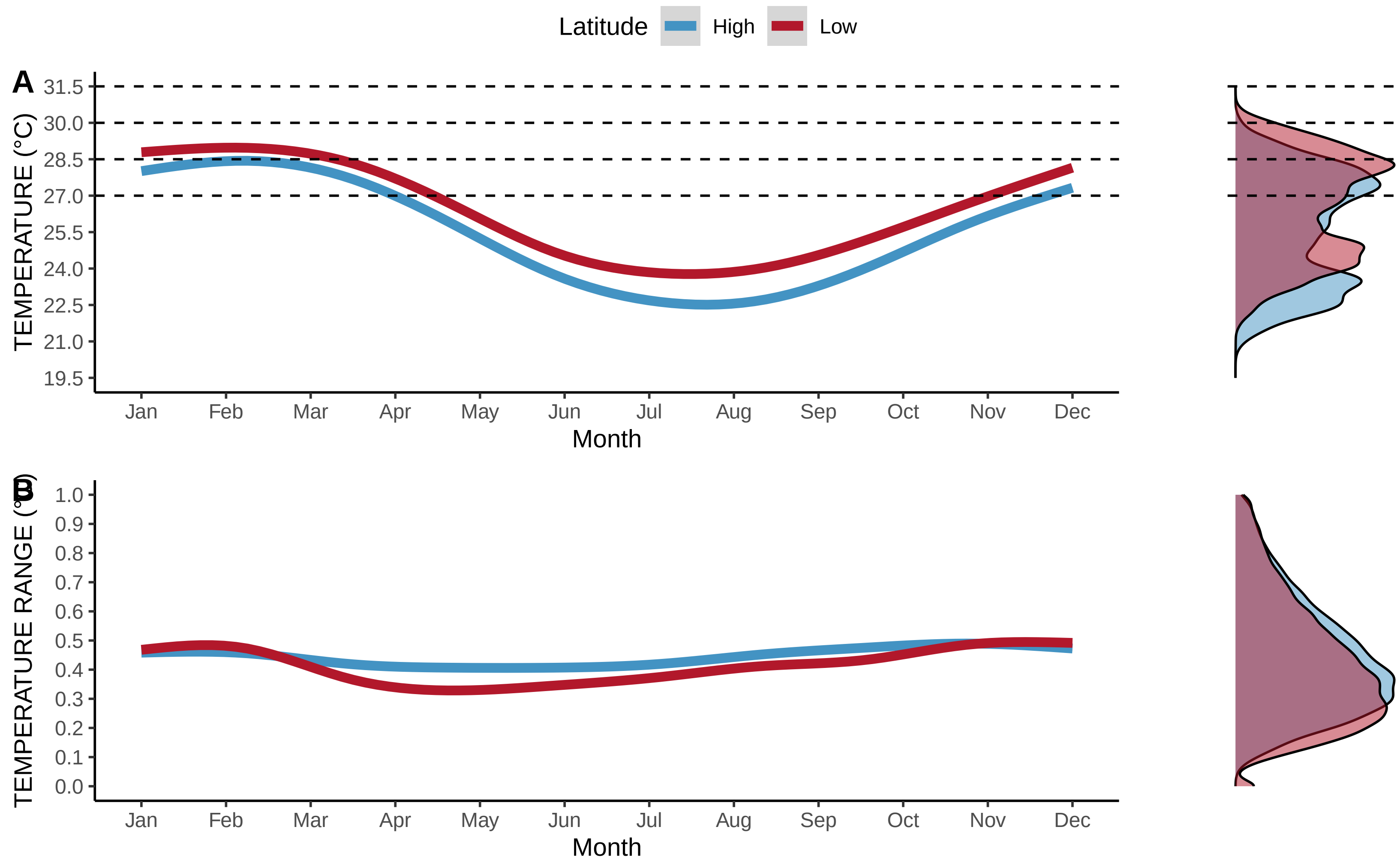

Supplemental figure 1: Seasonal temperature profile for reefs within the low− and high−latitude region of the Great Barrier Reef (see Stab.2 for list of reefs names). A) mean daily temperature and B) mean daily range are shown for both low− (solids red line) and high−latitudinal (dashed blue line) regions, as well as density plots identifying the most frequently experienced temperatures or ranges experienced. Data was obtained via the Australian Institute of Marine Science Temperature Logger dataset (AIMS 2020).

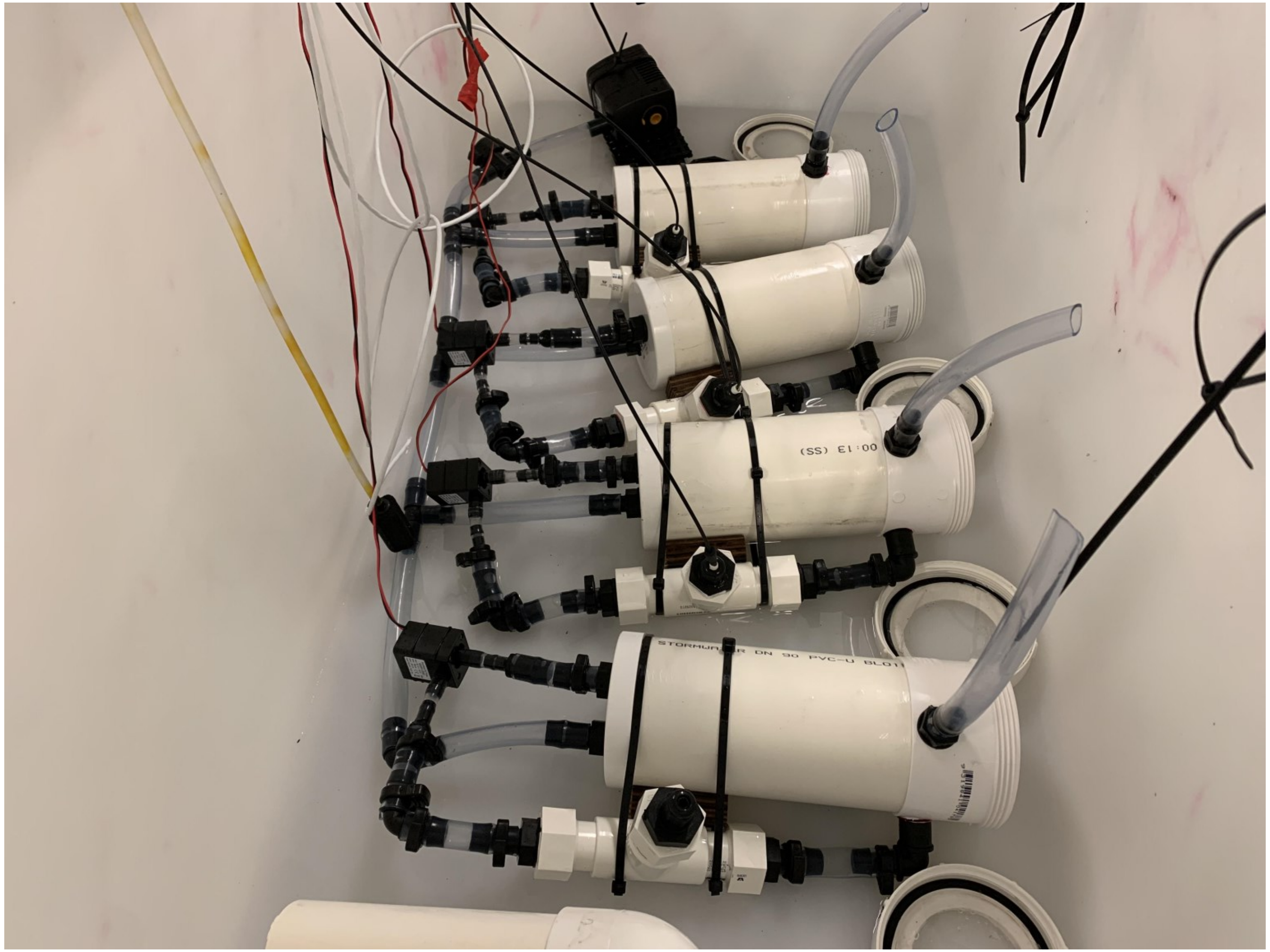

Supplemental figure 2: Respirometry chambers that were made in-house and used to measure oxygen consumption traits.

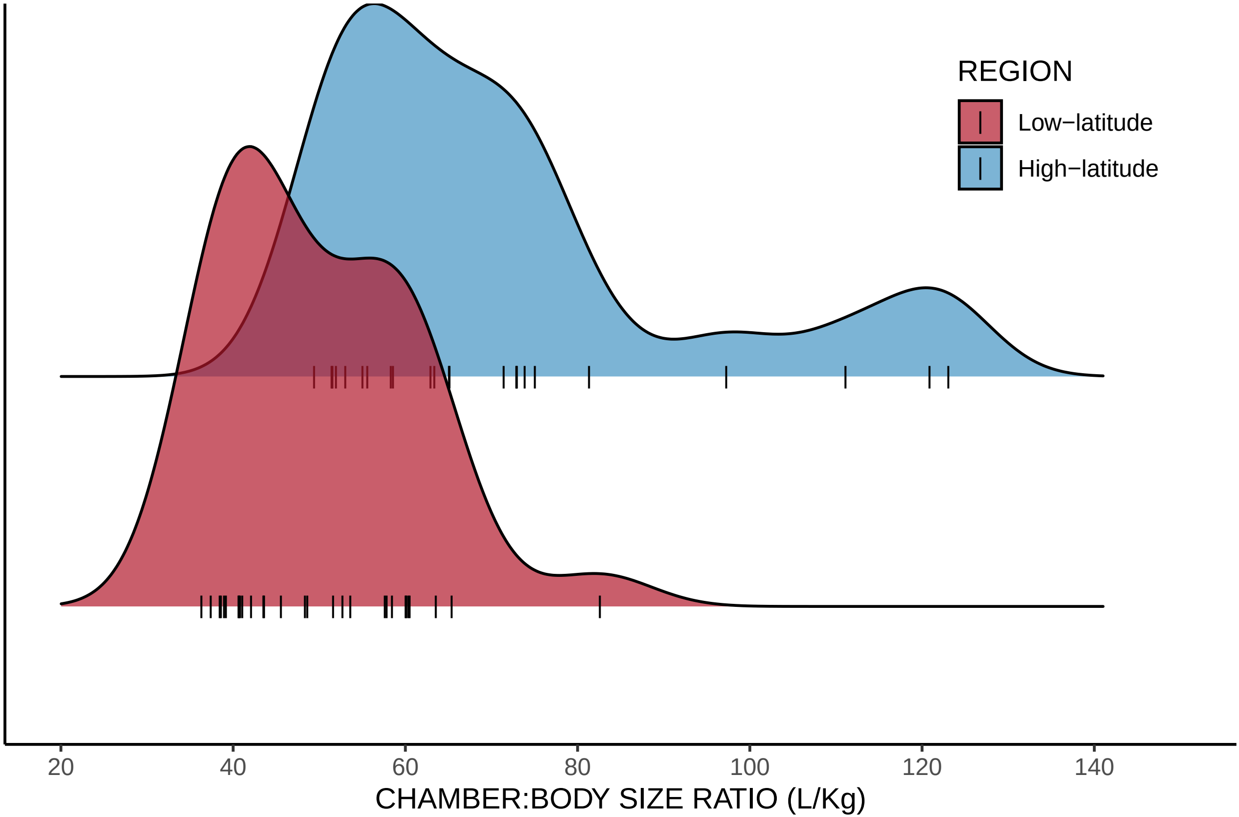

Supplemental figure 3: Density plots displayed fish body size to chamber ratios. Fish that were sampled for aerobic physiology from the low−latitude region are represented in red; fish from the high−latitude region are represent in blue.

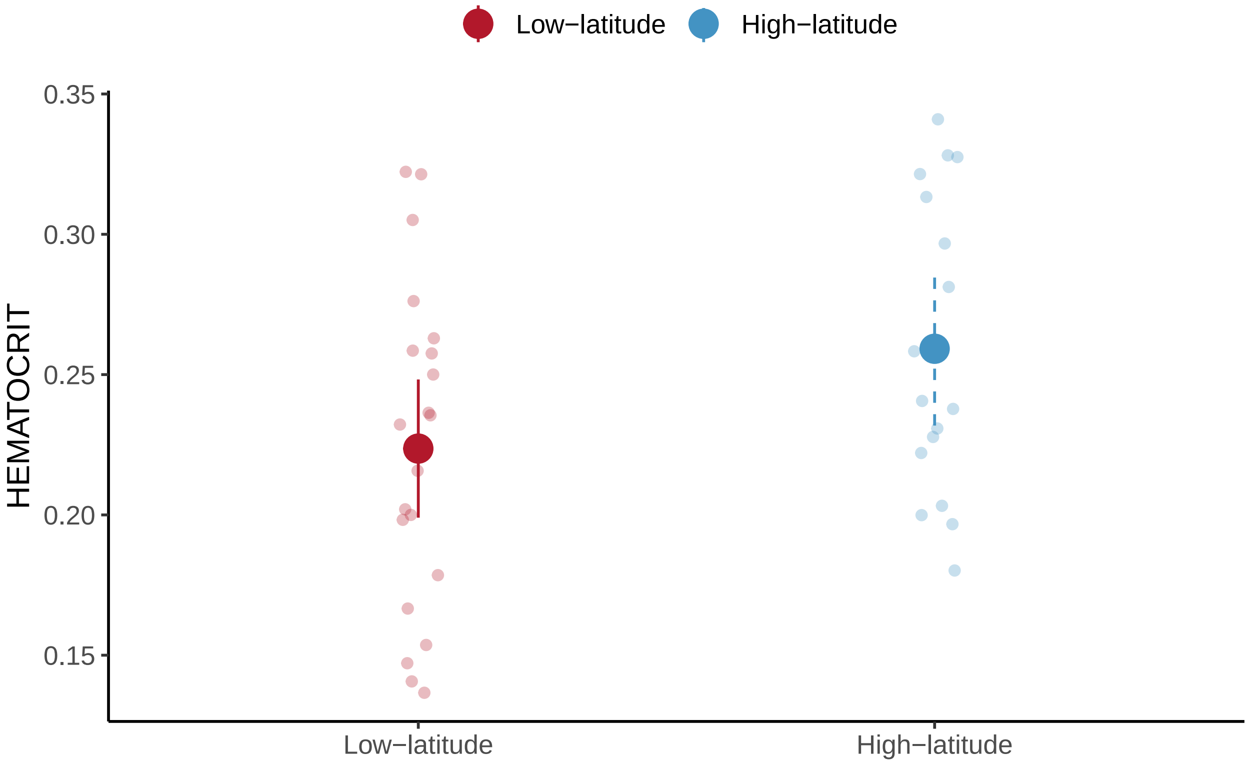

Supplemental figure 4: Comparison of hematocrit ratios, that were measured at 31.5°C, between low−(red) and high−latitudinal (blue) populations. No significant difference was observed between the different latitudes ($p = 0.058$). Solid (low−latitude) and dashed (high−latitude) lines represent 95% confidence intervals.

Supplemental table 1: Rationale for using difference physiological metrics that were examined in this study.

Trait	Rationale
**Metabolic traits**	
**Resting metabolic rate (MO_{2Rest})**	Baseline metabolic rate can be defined in different terms including, standard metabolic rate ($MO_{2standard}$) – rate of oxygen consumption when organisms exhibit minimal function activity (i.e., absence of voluntary muscle movement and digestion); and resting metabolic rate (MO_{2Rest}) – rate of oxygen consumption when organisms display minor activity in respirometer (i.e., minor cost of activity) (Chabot et al., 2016). In this experiment MO_{2rest} was used because *A. polyacanthus* spend most of the time using small fin movements to maintain position in the water column, making in more ecologically relevant for the study species in this experiment.
**Maximum metabolic rate (MO_{2max})**	Maximum metabolic rate (MO_{2max}) aims to quantify the maximum rate of oxygen consumption an organism can perform. MO_{2max} is typically determined by measuring oxygen consumption during exercise or immediately after (Clark et al., 2013).
**Absolute aerobic scope (AAS)**	Absolute aerobic scope (AAS) is obtained from subtracting MO_{2rest} from MO_{2max}. AAS provides an indication of oxygen consumption rates that can be achieved above baseline levels (Clark et al., 2013). Under the oxygen- and capacity limitations and thermal tolerance hypothesis (OCLTT), AAS is hypothesized to provide an indication of organism performance across a thermal performance curve (Pörtner et al., 2017; Pörtner and Farrell, 2008; Pörtner and Knust, 2007). However, the OCLTT does not appear to be universally applicable and remains contested within the literature (Lefevre et al., 2021). AAS (and therefore MO_{2rest} from MO_{2max}) have been widely used in physiological studies, including previous research on *A. polyacanthus* (Donelson et al., 2012; Donelson and Munday, 2012; Gardiner et al., 2010; Rummer et al., 2014).
**Immunocompetence traits**	
**Immunocompetence (Phytohemagglutinin; PHA)**	The phytohemagglutinin (PHA) skin-swelling test acts as an *in vitro s*urrogate metric for immunocompetence (Martin et al., 2006). PHA triggers the proliferation of leukocytes (primarily T-lymphocytes) upon injection, however, the overall function sense of test remains unclear (e.g., does double the swelling mean double the immunocompetence?; Martin et al., 2006). The use of PHA skin swelling test has been piloted in *A. polyacanthus* previously by Donelson and Yasutake (2024). Additionally, it has been used on other coral reef fish species including rabbitfish (i.e., *Siganus doliatus* and *S. lineatus*; LaMonica et al., 2021).

**Enzyme traits**	
**Lactate dehydrogenase (LDH)**	Lactate dehydrogenase (LDH) serves as an index for anaerobic glycolytic potential (Jayasundara et al., 2013). LDH is measured by monitoring the decrease of NADH in samples (using a spectrophotometer), as LDH is involved in converting NADH to NAD^+ (Jayasundara et al., 2013). An increase in LDH activity would indicate an increased reliance on anaerobic glycolysis to meet ATP demands (Ekström et al., 2017). LDH has been used to assess the response of organisms in several aquatic species including crown-of-throne sea stars (*Acanthaster* sp.;Lang et al., 2021), rainbow trout (*Oncorhynchus* mykiss; Pichaud et al., 2017), European perch (*Perca* fluviatilis; Ekström et al., 2017), emerald rockcod (*Gillichthys* mirabilis; Jayasundara et al., 2013), as well as several coral reef species including *Amphiprion melanopus*, *Lates calcarifer, Caesio cuning*, *Cheilodipterus quinquelineatus,* and *Acanthochromis polyacanthus* (Illing et al., 2020; Johansen et al., 2021).
**Citrate synthase (CS)**	Citrate synthase (CS) serves as an index for aerobic potential and a proxy for mitochondrial volume density (Illing et al., 2020). CS is measured by monitoring the production of citrate within samples (using a spectrophotometer). CS catalyzes the condensation of acetyl-CoA and oxaloacetate to product citrate during the first step of the citric cycle (Illing et al., 2020). CS has been used to assess aerobic metabolism in several species (*see species and references listed in LDH section above*)
**Hematological traits**	
**Hematocrit**	Hematocrit measurements refer to the percentage of the whole blood that is composed of packed red blood cells. Red blood cells are responsible for the transport of oxygen, therefore, in vertebrates the fraction of rec blood cells (found in whole blood) will partly determine the oxygen carrying capacity of blood within organisms (Gallaugher et al., 1995). Hemoglobin can also impact oxygen carrying capacity of blood within organisms (although hemoglobin was not analyzed in this study). According to the OCLTT hypothesis if oxygen capacity is limiting performance at higher temperatures, one approach organisms could use to acclimate to warmer conditions could be to increase oxygen carrying capacity of blood via increasing red blood cell concentration in the blood (Gallaugher et al., 1995). Hematocrit has been used to assess responses to warming temperatures in aquatic organisms previously including the Pacific spiny dogfish (*Squalus suckleyi*; Bouyoucos et al., 2023), *Caesio cuning*, and *Cheilodipterus quinquelineatus* (Johansen et al., 2021).

Supplementary table 2: Samples sizes of fish that were used from each population over the course of the experiment. $N_{all\ measurements}$ refers to fish that completed aerobic physiology and immunocompetence experiments at all testing temperatures (i.e., 27, 28.5, 30, and 31.5°C), in addition to having tissue and blood samples collected at the conclusion of the experiment.

Population	***Latitude***	***N***	***$N_{all\ measurements}$***
Sudbury Reef	Low-latitude	11	9
Tongue Reef	Low-latitude	8	6
Vlassof Cay	Low-latitude	10	6
Cockermouth Island	High-latitude	10	8
Keswick Island	High-latitude	6	4
Chauvel Reef	High-latitude	10	5
Total		***55***	***38***

Supplemental table 3: List of reefs from the Australian Institute of Marine Science Temperature Logger (Australian Institute of Marine Science (AIMS) 2020) dataset that were used to determine the thermal regime of low- and high-latitude regions. Only temperature loggers that were placed between 7-15m deep were sampled. Latitude and longitude are measured in decimal degrees.

Site	Subsite id	Series	Latitude	Longitude	Latitude
**Davies Reef**	2630	DAVSL1	-18.806	147.6686	Low-latitude
**Davies Reef**	3272	DAVAWSL2	-18.8313	147.634	Low-latitude
**Myrmidon Reef**	2726	MYRSL1	-18.2572	147.3813	Low-latitude
**Orpheus Island**	14957	ORPHCH2	-18.6017	146.4881	Low-latitude
**Rib Reef**	10200	RIBSL1	-18.4719	146.8788	Low-latitude
**John Brewer**	2723	JBRSL1	-18.6188	147.0815	Low-latitude
**Kelso Reef**	2620	KELSL1	-18.4221	146.9846	Low-latitude
**Dip Reef**	2633	DIPSL1	-18.3999	147.4519	Low-latitude
**Green Island, Qld**	2672	GRESL1	-16.7755	145.9803	Low-latitude
**Moore Reef**	2708	MORSL1	-16.8477	146.2358	Low-latitude
**Arlington Reef**	2738	ARLSL1	-16.6461	146.1107	Low-latitude
**Frankland Islands**	3177	FRNKSL2	-17.227	146.0904	Low-latitude
**Pandora Reef**	3247	PANSL1	-18.8168	146.4346	Low-latitude
**Pandora Reef**	10016	PANSL2	-18.8116	146.43	Low-latitude
**Snapper Island North**	3291	SNAPISSL1	-16.2919	145.4965	Low-latitude
**Chicken Reef**	2635	CHISL1	-18.6521	147.7217	Low-latitude
**Flinders Reef**	14617	FLINDERSSL2	-17.7123	148.4509	Low-latitude
**Havannah Island**	14975	HAVSL2	-18.8315	146.5374	Low-latitude
**Thetford Reef**	10635	THETSL1	-16.7988	146.1981	Low-latitude
**Thetford Reef**	13535	THETFL1	-16.7995	146.1951	Low-latitude
**19-131 Reef**	12196	19131SL1	-19.7662	149.3802	Low-latitude
**19-131 Reef**	15438	19131SL3	-19.7728	149.376	Low-latitude
**19-138 Reef**	12198	19138SL1	-19.8069	149.4305	Low-latitude
**Rebe Reef**	12208	REBESL1	-19.7919	150.1612	Low-latitude
**St Crispin Reef**	12421	STCRISPSL1	-16.0713	145.8453	Low-latitude
**Opal Reef**	12579	OPALSL1	-16.2034	145.9065	Low-latitude
**Peart Reef**	12657	PEARTSL1	-17.4665	146.4031	Low-latitude
**Feather Reef**	12655	FEATHSL1	-17.5179	146.3905	Low-latitude
**Hedley Reef**	13638	HEDLSL1	-17.2251	146.479	Low-latitude
**McCulloch Reef**	12777	MCCULLSL1	-17.2841	146.4796	Low-latitude
**Farquharson Reef**	13637	FARQSL1	-17.7912	146.5324	Low-latitude
**Taylor Reef**	13636	TAYLORSL1	-17.8107	146.5716	Low-latitude
**Roxburgh Reef**	12761	ROXSL1	-18.4283	147.0593	Low-latitude
**Helix Reef**	12656	HELIXSL1	-18.6201	147.2976	Low-latitude
**Grub Reef**	12758	GRUBSL1	-18.6216	147.4327	Low-latitude
**Knife Reef**	12775	KNIFESL1	-18.5711	147.5772	Low-latitude
**Hastings Reef**	13898	HASTSL1	-16.4995	146.0223	Low-latitude
**Agincourt Reef Number 1**	14835	AG1SL1	-16.0384	145.8688	Low-latitude
**Pine Island**	3080	PINESL1	-20.378	148.8884	High-latitude
**Seaforth Island**	3087	SEAFSL1	-20.4685	149.0389	High-latitude

**Shute and Tancred Islands**	3679	SHTANSL1	-20.3009	148.7983	High-latitude
**20-104 Reef**	12204	20104SL1	-20.0294	149.6951	High-latitude
**Border Island**	12210	BORDERSL1	-20.1819	149.0377	High-latitude
**21-245 Reef**	12398	21245SL1	-21.2965	152.4465	High-latitude
**21-062 Reef**	12422	21062SL1	-21.0266	150.8538	High-latitude
**21-139 Reef**	12435	21139SL1	-21.4547	151.4654	High-latitude
**21-187 Reef**	12436	21187SL1	-21.4054	151.6384	High-latitude
**Tern Reef**	12763	TERNSL1	-20.899	150.0289	High-latitude
**Penrith Reef**	12760	PENRITHSL1	-21.0032	149.8875	High-latitude
**21-591 Reef**	12757	21591SL1	-21.0266	150.3807	High-latitude
**21-550 Reef**	12586	21550SL1	-21.9612	152.3168	High-latitude
**Gannet Cay Reef**	13639	GANNETSL1	-21.9758	152.4797	High-latitude

Supplemental table 4: Information pertaining to statistical models that were run to identify differences between low- and high-latitude reefs for aerobic physiology, enzyme, immunocompetence, and hematocrit metrics. Formulas in the table are expressed using R-language formula notation.

Response variable	Distribution	Link	Interactions	Covariates	Random effects
**Aerobic physiology**					
▪ Resting oxygen consumption	gaussian	identity	Latitude*poly(Temperature, 2)	Fish Mass (centred)	(1|Fish_id)
▪ Maximum oxygen consumption	gaussian	identity	Latitude*poly(Temperature, 2)	Fish Mass (centred)	(1|Fish_id)
▪ Absolute aerobic scope	gaussian	identity	Latitude*poly(Temperature, 2)	Fish Mass (centred)	(1|Fish_id)
**Enzymes**					
▪ Lactate dehydrogenase	gaussian	identity	Latitude*poly(Temperature, 2)	Tissue Mass (centred)	(1|Fish_id)
▪ Citrate synthase	gaussian	log	Latitude*poly(Temperature, 2)	Tissue Mass (centred)	(1|Fish_id)
▪ Lactate dehydrogenase: citrate synthase	gaussian	identity	Latitude*Temperature	Tissue Mass (centred)	(1|Fish_id)
**Immunocompetence**	gamma	log	Latitude*poly(Temperature, 3)	-none-	(1|Population/Fish_id)
**Hematocrit**	gaussian	identity	Latitude	-none-	-none-

Supplemental table 5: Mean phytohemagglutinin measurements for all fish sampled at the four different treatment temperatures (i.e., 27, 28.5, 30, and 31.5°C). Differences in sample size are due to mortality events that fish experienced during the experiment. Measurements between different temperature treatments should not be treated as pairwise comparisons due to the differences in sample size.

Temperature (°C)	Sample size	Mean pre-injection width (mm)	Mean post-injection width (mm)	Mean Δ absolute difference (mm)	Mean Δ percent difference (%)
**27**	52	4.24	4.49	0.25	5.87
**28.5**	50	4.34	4.67	0.34	7.93
**30**	43	4.59	4.75	0.16	3.75
**31.5**	39	4.53	4.59	0.055	1.28
